# The potential impact on tuberculosis of interventions to reduce undernutrition in the WHO South-East Asian Region: a modelling analysis

**DOI:** 10.1016/j.lansea.2024.100423

**Published:** 2024-05-16

**Authors:** Sandip Mandal, Vineet Bhatia, Anurag Bhargava, Suman Rijal, Nimalan Arinaminpathy

**Affiliations:** a John Snow India, New Delhi, India; b World Health Organization, South-East Asia Regional Office, New Delhi, India; c Department of General Medicine, Yenepoya Medical College, Karnataka, India; d MRC Centre for Global Infectious Disease Analysis, Imperial College London, London, UK

**Keywords:** Tuberculosis, Undernutrition, Preventions, WHO South-East Asian Region, Mathematical model

## Abstract

**Background:**

Undernutrition is a major risk factor for TB incidence in the WHO South-East (SE) Asia Region. We examined the potential impact of addressing undernutrition as a preventive measure, for reducing TB burden in region.

**Methods:**

We developed a deterministic, compartmental mathematical model, capturing undernutrition and its associated excess risk of TB, amongst countries in the Region. We simulated two types of interventions: (i) nutritional rehabilitation amongst all close contacts of TB patients, and (ii) an illustrative, population-wide scenario where 30% of people with undernutrition would be nutritionally rehabilitated each year. We also simulated this impact with additional measures to improve the TB care cascade.

**Findings:**

The impact of nutritional interventions varies by country. For example, in India nutritional rehabilitation of 30% of undernourished population each year would avert 15.9% (95% Uncertainty Intervals (UI) 11.8–21.3) of cumulative incidence between 2023 and 2030, contrasting with 4.8% (95% UI 2.9–9.5) for Bhutan, which has only 10.9% prevalence of undernutrition. Reductions in cumulative mortality range from 11.6% (95% UI 8.2–17.1) for Bhutan, to 26.0% (95% UI 22.4–30.8) for India. Comparable incremental reductions in TB burden arise when combined with measures to improve the TB care cascade. Overall, nutritional interventions in the general population would increase incidence reductions by 2–3 fold, and mortality reductions by 5–6 fold, relative to targeting only contacts.

**Interpretation:**

Nutritional interventions could cause substantial reductions in TB burden in the Region. Their health benefits extend well beyond TB, underlining their importance for public health.

**Funding:**

None.

**Research in context**

**Evidence before this study**

Undernutrition and its related immunodeficiency has long been recognised as a key determinant of tuberculosis (TB) incidence and mortality. It is especially prevalent in the WHO South-East Asian Region, with WHO estimating a population-attributable fraction of around 50% in the Region. Previous modelling work, focused on India, highlighted the strong impact on tuberculosis (TB) that could arise from interventions to address undernutrition. Another analysis showed how such interventions would be cost-effective, even when modelling only direct health benefits (i.e. without considering transmission reductions). In the absence of trial data, both studies had to make assumptions for the effectiveness of nutritional interventions in reducing TB incidence and mortality. The recent RATIONS trial in India was an important step towards addressing this data gap, demonstrating, for example, how nutritional rehabilitation under programmatic conditions could reduce incidence of all forms of TB amongst close contacts of TB patients by nearly 40%, and microbiologically confirmed pulmonary TB by nearly 50%.

**Added value of this study**

For the first time, we incorporated results from the RATIONS trial into a mathematical model of TB transmission dynamics, to estimate the potential incidence and mortality reductions that could arise from scaling it up to countrywide coverage. While previous analyses focused on India, we expanded the scope of the analysis to include 10 of the 11 countries in the South-East Asia Region, representing a range of contexts in terms of the burden of TB, and the prevalence of undernutrition. Our analysis shows, for example, that reductions in cumulative incidence between 2023 and 2030 arising from nutritional supplementation amongst all close contacts of notified TB patients would range from 0.3% (95% UI 0.05–1.4) in Bhutan to 11.9% (95% UI 8.6–16.5) in Timor-Leste. Expanding the intervention to cover 30% of all people with undernutrition would enhance this impact substantially, from 4.8% (95% UI 2.9–9.5) in Bhutan to 15.9% (95% UI 11.8–21.3) in India.

**Implications of all the available evidence**

Addressing undernutrition would have an important impact on TB incidence and mortality, particularly amongst countries in the WHO South-East Asia Region, where undernutrition plays a particularly important role in driving TB and its outcomes.

## Introduction

The World Health Organization (WHO) End TB (tuberculosis) Strategy, in alignment with the United Nations (UN) Sustainable Development Goals, aim to end TB globally.^1^ The WHO South-East (SE) Asia Region bears 4.8 million new cases, or nearly half of the global tuberculosis incidence annually, and accounts for more than 750,000 deaths due to the disease annually.^2^ As the world prepares to reclaim its lost ground in progress towards ending TB in the post-COVID-19 era, the focus is now on the SEA Region with the highest burden of TB among all WHO regions.

Undernutrition at the population level is one of the key determinants of TB in the SE Asia Region, and recent estimates published by WHO suggest that more than 1 million new TB cases (nearly 20% of incidence) annually are attributable to undernutrition.^2^ Undernourished people are estimated to be three times more likely to develop the disease^1^^,^^3^ as undernutrition is the commonest cause of secondary immunodeficiency and affects both innate and adaptive immunity. Undernutrition in patients can be disease-related undernutrition or worsening of pre-existing chronic undernutrition related to TB disease. Tuberculosis can result in secondary wasting due to increased metabolic demands and anorexia. Undernourished patients with tuberculosis have delayed recoveries and higher mortality rates,^4^ while their nutritional status usually improves with appropriate tuberculosis chemotherapy. A low body mass index (BMI) puts TB patients at higher risk of severe disease, delayed sputum conversion, drug-induced hepatotoxicity, malabsorption of antituberculosis drugs, and relapse after cure.^2^ Although the negative effects of TB on the body weight of patients and the positive effects of a good diet are known, the efforts to address nutrition among TB patients have been variable across the SEA Region.

The WHO End TB Strategy targets pertain to reduction of TB incidence, reduction of TB mortality, and zero catastrophic costs due to the disease. Recent evidence from the RATION trials shows that nutritional support to underweight patients with TB can reduce TB mortality significantly,^5^ while similar support to close contacts of TB patients can substantially reduce incidence amongst these contacts.^6^ Overall, there is a need to reduce the prevalence of undernutrition at the population level, especially in those who are in close contact or suffer most from infectious forms of TB.^7^^,^^8^ Addressing TB and accelerating efforts towards ending TB would need food security for TB patients, as well as those at risk of developing TB. This would need multisectoral and multidisciplinary approaches within countries and internationally and could constitute a key target for multisectoral efforts to end TB.

Here, we sought to use mathematical modelling to estimate the reductions that could be achieved in TB incidence and mortality, with accelerated efforts for nutrition rehabilitation amongst countries in the Region. We modelled 10 out of the 11 countries in the Region with the exception of the Maldives, as this is a country with low levels of malnutrition, low TB burden, and only 4% prevalence of TB infection (Table 1). Previous work estimated the potential impact of nutritional interventions on the TB epidemic in India,^10^ as well as its cost-effectiveness.^11^ Our current work builds on this analysis, incorporating updated estimates of TB burden in India in the wake of COVID-19 related disruptions, as well as results from a recent trial of nutritional interventions under programmatic conditions in India,^12^ and expanding this analysis to the other countries in the SE Asian Region.

**Table 1:** List of countries in the SEA Region, and data used as calibration targets.

Country	Incidence per 100,000 (2019)	Mortality per 100,000 (2019)	Notification per 100,000 (2019)	Prevalence of TB infection (2019, proportion of population)	Prevalence of underweight among adults, BMI <18.5 (age-standardized estimate) (%) in 2016
Bangladesh	221 [161–291]	24 [15–34]	180	0.26 [0.23–0.28]	21.5 [16.8–26.7]
Bhutan	165 [126–208]	18 [12–26]	133	0.19 [0.17–0.21]	10.9 [6.6–16.2]
DPR Korea	513 [446–584]	71 [48–95]	396	0.60 [0.53–0.66]	6.6 [3.1–12.2]
India	214 [146–295]	32 [30–34]	176	0.25 [0.23–0.28]	23.6 [19.9–27.6]
Indonesia	312 [285–341]	34 [32–36]	210	0.36 [0.33–0.40]	12.9 [9.3–16.9]
Maldives	36 [28–46]	2.1 [1.9–2.3]	29	0.04 [0.03–0.05]	9.2 [5.7–13.8]
Myanmar	322 [212–454]	36 [21–54]	254	0.38 [0.34–0.41]	14.6 [10.4–19.5]
Nepal	238 [141–359]	58 [32–92]	112	0.28 [0.25–0.31]	16.8 [12.6–21.3]
Sri Lanka	64 [47–83]	3.5 [3.1–3.8]	40	0.07 [0.06–0.08	14 [9.8–18.8]
Thailand	150 [114–191]	14 [10–18]	129	0.18 [0.16–0.19]	8.6 [5.6–12.3]
Timor-Leste	498 [322–711]	88 [52–133]	328	0.58 [0.52–0.64]	16.1 [11.2–21.7]

As described in the supporting information, likelihood terms were constructed to capture the central estimates and uncertainty intervals for each of the data elements shown. For notifications, for the purpose of constructing likelihood functions we assumed a range of ± 10% of the values shown. Data for the prevalence of adult undernutrition was taken from the Global Health Observatory.^9^ As described in the main text, the Maldives were exempted from this analysis, owing to the low burden of TB and undernutrition.

## Methods

### Model overview

**Fig. 1:**
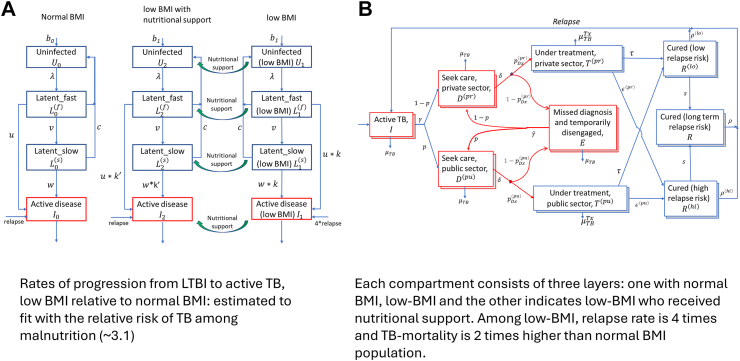
**Schematic illustration of the model structure.** The figure shows model components relating to nutritional status. Infectious compartments are shown in red, and compartments constituting the prevalence of TB infection are all those labelled ‘latent fast’ and ‘latent slow’. Additional model structure, not shown here for simplicity, include the TB care cascade (diagnosis and treatment, stratified by public and private sectors), and factors such as reinfection and endogenous relapse. The left–hand plot illustrates how states preceding TB disease are stratified by nutritional status. The right–hand plot shows model compartments relating to subsequent stages in the TB cascade, with all stratifications relating to nutritional status shown as ‘layers’ on each compartment.

We developed a deterministic, compartmental model of TB transmission for each country in the Region, with structure illustrated schematically in Fig. 1. The model captures essential features of TB natural history and epidemiology, including the important role played by the private healthcare sector in many countries in the Region. For simplicity, the model does not incorporate age structure, gender differences in TB burden, or the difference between pulmonary and extrapulmonary TB. A key component of the model is that compartments relating to TB infection are stratified by nutritional status, in particular distinguishing ‘low BMI’ (as a reference point, cut-off of BMI <18.5 has been taken for the purpose^13^) from ‘normal BMI’ status and ‘low BMI with rehabilitation’. We assumed that the effect of undernutrition is to amplify the rates at which TB-infected individuals progress to active disease, chosen (as described below) to match the relative risk of TB amongst those with low BMI, compared to those with normal BMI. We assumed further that undernutrition increases mortality rates of TB patients while on treatment, drawing from programmatic data to inform the associated mortality parameters in the model. As described below, we drew from recent evidence from a cluster-randomized trial amongst household contacts in India,^12^ for the impact of rehabilitation, on the risk of developing active TB, as well as the hazard of TB mortality before and during treatment.

### Data and calibration

The COVID-19 pandemic caused widespread disruptions to TB services and worsening of social determinants, likely resulting in increases in TB incidence and mortality due to an accumulation in the burden of undetected, untreated TB.^14^ To capture pre-COVID-19 estimates of TB burden, we calibrated the model illustrated in Fig. 1 separately to each of the 10 modelled countries in the Region. Table 1 lists specific calibration data that was used for each country, including incidence and mortality in 2019, and the prevalence of undernutrition. To capture how the TB epidemic in each country reacted to COVID-19 related disruptions, we followed the same modelling approach as that used in the recent WHO global TB report^2^: assuming that reductions in notifications during the COVID-19 pandemic were attributable to reductions in TB diagnosis, we adjusted the rate of presentation to care on a quarterly basis so that TB treatment initiations in the notifying sector would match quarterly notifications reported to WHO between January 2020 and October 2022 (the date of the latest available data, provisional for 2022 at the time of undertaking this analysis).^2^

Calibration was performed using Bayesian Markov Chain Monte Carlo (MCMC). In particular, we constructed likelihood functions for each of the calibration targets shown in Table 1. We used log-normal distributions for population rates (such as incidence), and beta distributions for proportions (such as the prevalence of TB infection), with distributional parameters chosen to capture central estimates and uncertainty intervals in the data. Given a lack of evidence from the literature to inform direct (i.e. non-modelled) estimates of calibration parameters, we assumed uniform priors for all such parameters, within plausible ranges (see Table S1 in the supporting information for specifications of these uniform priors). We then constructed a posterior density as a product of these likelihood functions and uniform priors. We sampled from this posterior density using an adaptive algorithm.^15^ After discarding the burn-in and ‘thinning’, we drew 250 samples for simulation (see supporting information for sensitivity analysis to the number of samples). For all model projections, we denoted the interval between the 2.5th and 97.5th percentiles as the 95% uncertainty intervals (UIs). We estimated the central estimate using the 50th percentile.

### Modelling interventions

We modelled two scenarios of nutritional supplementation: first, a scenario consistent with scaling up the recent RATIONS trial in India.^5^^,^^12^ That is, a nutritional package for all contacts of index cases, regardless of nutritional status, but with enhanced support for those with low BMI^12^ or other pre-defined indications of undernutrition. The RATIONS trial showed strong reductions in cohort incidence even amongst contacts with normal BMI and above. Accordingly, we modelled the impact of reducing incidence amongst all household contacts by 40%, consistent with the overall incidence reductions observed in the RATIONS trial. To do so, we borrowed from a previously developed approach for modelling preventive interventions amongst household contacts, that was also developed for countries in the Region.^16^

For the second intervention scenario, we assumed that nutritional support would extend beyond household contacts, to address undernutrition in the general population. As an example of ambitious levels of population coverage, we assumed an intervention that provides nutritional rehabilitation to 30% of undernourished people each year: we modelled this intervention as shifting individuals from ‘low’ BMI to ‘low BMI with rehabilitation’ status in Fig. 1, assuming that this intervention would be scaled up in a linear way between 2023 and 2025. Further, we assumed that those having ‘low BMI with rehabilitation’ status would show the same incidence reductions as amongst undernourished contacts in the RATIONS study, i.e. of 40%.^12^

For each country, we additionally modelled the potential impact of nutritional supplementation under two scenarios: when acting on its own, and when acting in combination with programmatic improvements including accelerated case-finding and improved treatment. In previous work, we modelled the impact of different types of programmatic improvement separately and in combination.^7^^,^^8^ Because the focus of the current work is on the incremental impact of nutritional interventions, we took a simpler approach, modelling programmatic improvements as a combination of: (i) a 30% reduction in the average delay to careseeking, and (ii) an increase to 90% of diagnosed patients being notified in all sectors, both interventions consistent with modelling for the recent Global Plan to End TB.^7^

### Role of the funding source

Not applicable.

## Results

Figures S2 and S3 in the supporting information show results of model calibration for each of the countries in the Region. Figures S5 and S7 shows MCMC traces for each country, illustrating well-mixed calibration, while Figures S4 show the results of matching to notifications during the COVID-19 pandemic. These figures illustrate that country-specific models capture the calibration targets, as well as the extent of COVID-19 related disruptions to TB services.

**Fig. 2:**
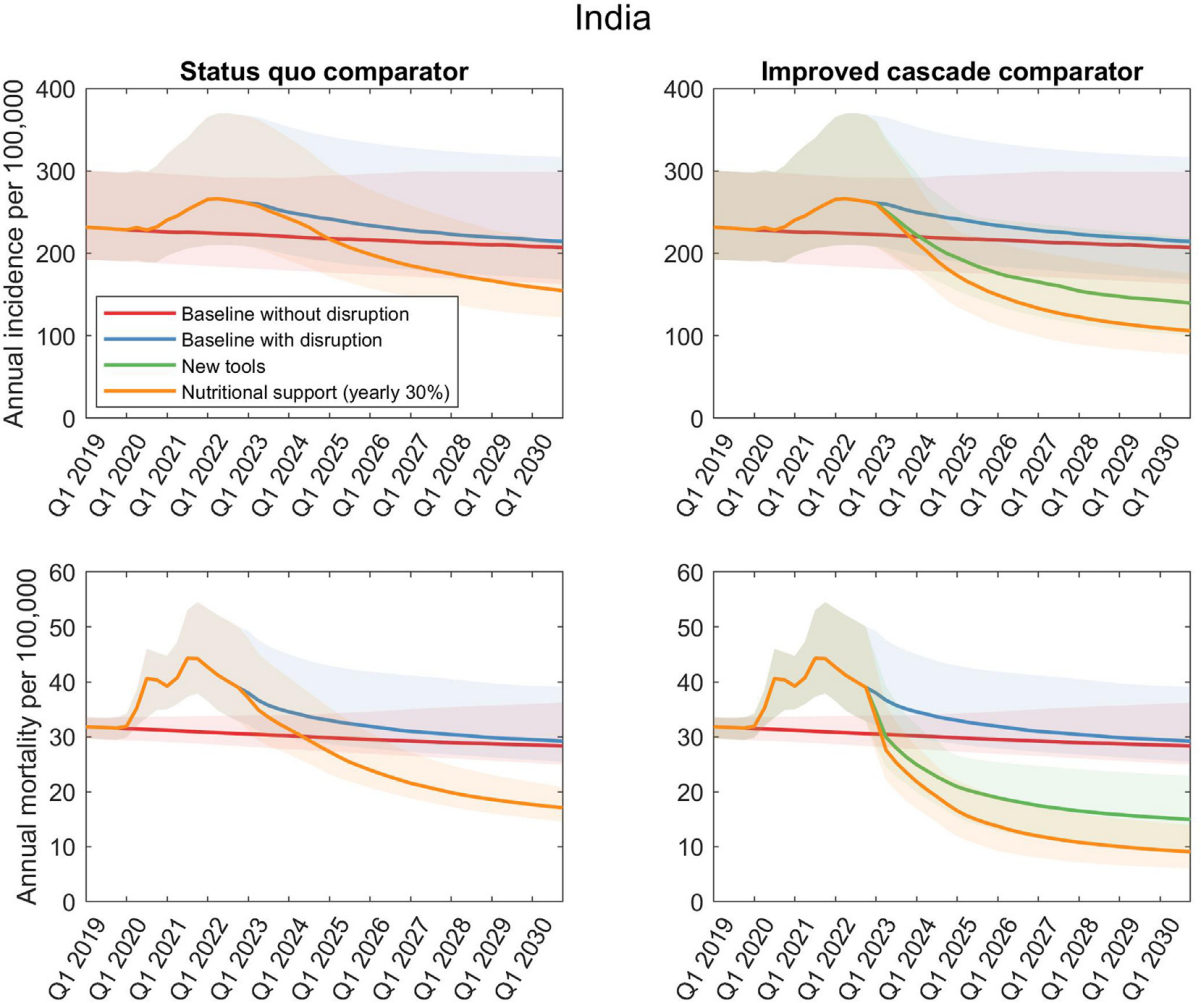
**Impact of nutritional interventions in the illustrative example of India.** As shown in Table 1, India is estimated to have a 23.6% prevalence of undernutrition in the adult population. Here, figures in the upper row show projections for incidence, taking account of COVID-related disruptions in India, while figures in the lower row show projections for mortality. The left-hand column shows the effect of nutritional interventions acting alone, while the right-hand column shows the effect of these interventions when acting in combination with measures to improve the TB care cascade.

Fig. 2 shows illustrative results, on TB incidence and mortality, of nutritional support. The Figure takes India as an illustrative example, the country with the highest absolute burden of TB globally, as well as having a substantial contribution to incidence from undernutrition. A nutritional intervention amongst contacts alone would reduce annual incidence rates at the country level by 5 .7% (95% UI 3.7–10.7) by 2030, relative to 2015, and annual mortality by 6.2% (95% UI 4.5–11.2). This impact would be substantially increased if nutritional rehabilitation is extended to the general population: where such an intervention reaches 30% of undernourished people per year, it would reduce the annual TB incidence rate by 23.6% (95% UI 16.6–44.1) and annual mortality by 35.5% (95% UI 28.6–58.4) between 2015 and 2030, relative to a scenario of no nutritional intervention. When combined with measures to improve TB services in these settings, nutritional support interventions are able to reduce annual incidence by a further 13.8% (95% UI 9.2–19.2) and annual mortality by a further 17.1% (95% UI 10.2–25.1) relative to a scenario of improved TB services alone.

**Fig. 3:**
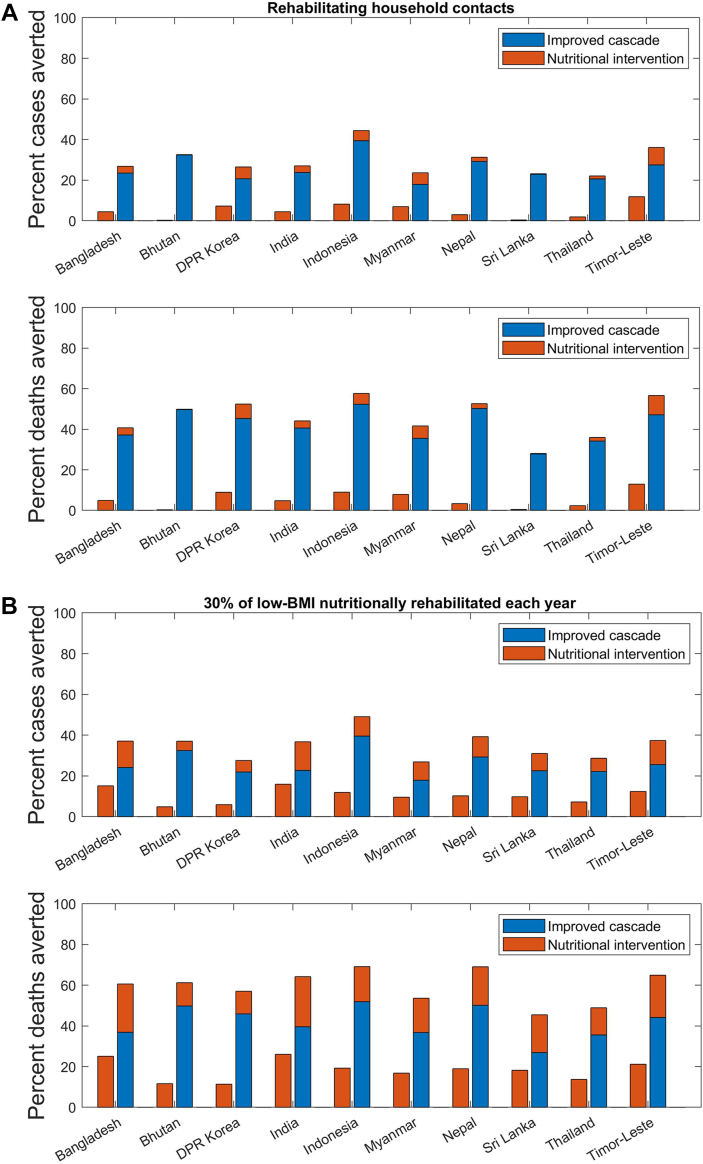
**Summary of impacts across high-burden countries in the Region.** Upper two panels (A) show TB burden averted, i.e. percent reduction in cumulative cases and deaths, in a scenario where nutritional support is provided to household contacts of TB patients. Lower two panels (B) show TB burden averted in a scenario where nutritional support is provided at the population level, even amongst those without known exposure to TB.

Fig. 3 summarises these impacts across countries in the Region, showing heterogeneity in potential impact. For example, potential cumulative incidence reductions from nutritional support interventions alone vary from 4.8% (Bhutan) to 15.9% (India). Generally, this range of impacts can be explained by the prevalence of undernutrition in the population, being substantially higher in India (around 24%) than in Bhutan (around 11%). As sensitivity analysis, Figure S9 in the supporting information illustrates—in the example of India—how the impact of nutritional supplementation in the general population would vary, with different levels of coverage.

## Discussion

It has been widely recognised that population-level prevention of TB will play a critical role in ambitious targets for the global response towards ending TB.^6^, ^7^, ^8^ Recent developments raise hope for an effective TB vaccine,^17^ but to pass Phase III trials and complete licensure will take years. In this context, our work highlights the important contributions that could be made through addressing undernutrition, a major determinant of TB in the WHO SE Asia Region. In particular, the impact of nutritional support interventions varies by country depending on the existing prevalence of undernutrition, with the potential incidence reductions between 2015 and 2030 ranging from 6% (95% UI 3.6–11.9) in Bhutan to 26.3% (95% UI 14.2–51.9) in Indonesia.

It is important to note that the public health rationale for such interventions extends well beyond TB: undernutrition has a range of adverse downstream effects on individual health, including on cognitive development, growth, heart and brain function, muscle mass and strength,^18^ and susceptibility to a range of infections including acute viral infections and diarrheal diseases.^19^ Early life nutrition and low birth weight are also linked to higher risk of non-communicable diseases in adulthood.^20^ Nearly 45% of all childhood deaths under five years of age are attributable to undernutrition.^21^ Thus, despite current uncertainties about the extent to which nutritional interventions might impact TB, the rationale for such interventions has a far wider scope and weight than TB. Moreover, there are potential benefits of nutritional rehabilitation not captured by our study: for example, the potential to improve adherence to TB treatment; uptake of preventive therapy amongst close contacts with TB infection; and to promote care-seeking amongst those with undiagnosed symptoms. Further evidence on the potential effects of nutritional rehabilitation on all of these factors will be invaluable in quantifying the full benefits for TB, of nutritional rehabilitation.

Our results also highlight the important impact that could arise, when nutritional supplementation is combined with other interventions (Fig. 2, and Table 2, Table 3). Measures such as private sector engagement are playing a critical role in increasing reporting from the private healthcare sector, amongst many countries in the Region.^8^ By doing so, they are also facilitating access to interventions such as nutritional supplementation, by the contacts of patients managed by the private sector. Thus, our results highlight synergies between these interventions and nutritional supplementation, that go deeper than a simple superposition of their independent effects.

**Table 2:** Summary of incidence impact in each country in the SE Asia Region by nutritional support.

Cases averted (2023–2030)	Strategy	Status quo comparator		Improved TB cascade comparator	
		Pct reduction in cumulative incidence (2023–2030)	Incremental incidence reductions in 2030 relative to 2015 attributable to nutritional interventions	Pct reduction in cumulative incidence (2023–2030)	Incremental incidence reductions in 2030 relative to 2015 attributable to nutritional interventions
Bangladesh	Household contacts	4.5 [3.6–5.9]	5.8 [3.9–11.6]	3.3 [2.1–4.3]	2.7 [1.4–4.2]
	30% coverage of low-BMI population	15.1 [11.3–20.3]	23.4 [14.9–45.8]	12.9 [9.8–17.3]	12.7 [7.5–19.7]
Bhutan	Household contacts	0.3 [0.05–1.4]	0.3 [0.04–1.5]	0.2 [0.03–0.8]	0.09 [0.01–0.5]
	30% coverage of low-BMI population	4.8 [2.9–9.5]	6.0 [3.6–11.9]	4.6 [2.8–8.0]	3.1 [1.9–6.2]
DPR Korea	Household contacts	7.3 [4.2–13.8]	10.2 [5.4–22.2]	5.8 [2.8–10.4]	4.9 [1.0–9.5]
	30% coverage of low-BMI population	5.9 [3.4–11.4]	10.0 [5.8–23.8]	5.6 [2.9–10.3]	5.9 [1.5–11.6]
India	Household contacts	4.5 [3.2–5.9]	5.7 [3.7–10.7]	3.3 [1.9–4.2]	2.7 [1.3–4.0]
	30% coverage of low-BMI population	15.9 [11.8–21.3]	23.6 [16.6–44.1]	14.0 [11.5–17.1]	13.8 [9.2–19.2]
Indonesia	Household contacts	8.2 [5.7–11.1]	14.5 [7.9–30.0]	5.0 [2.8–7.1]	3.7 [1.4–7.5]
	30% coverage of low-BMI population	11.9 [8.3–15.6]	26.3 [14.2–51.9]	9.5 [6.9–12.7]	9.3 [3.8–16.0]
Myanmar	Household contacts	7.0 [5.4–11.4]	8.9 [6.3–23.3]	5.7 [4.2–9.0]	4.9 [3.2–11.4]
	30% coverage of low-BMI population	9.6 [6.9–13.6]	14.8 [9.9–33.8]	9.0 [6.8–12.7]	9.7 [6.8–18.9]
Nepal	Household contacts	3.0 [2.1–4.0]	3.7 [2.2–6.6]	2.1 [1.3–2.8]	1.6 [0.6–2.8]
	30% coverage of low-BMI population	10.3 [4.7–15.1]	16.9 [6.9–29.8]	10.0 [6.7–13.4]	9.4 [4.9–16.6]
Sri Lanka	Household contacts	0.4 [0.2–0.6]	0.4 [0.3–0.7]	0.3 [0.1–0.5]	0.2 [0.07–0.5]
	30% coverage of low-BMI population	9.8 [7.5–12.9]	13.3 [9.5–19.2]	8.5 [6.4–11.2]	7.3 [4.6–11.5]
Thailand	Household contacts	1.9 [1.3–2.8]	2.3 [1.3–3.9]	1.5 [0.7–2.2]	1.2 [0.4–2.1]
	30% coverage of low-BMI population	7.2 [4.8–11.3]	10.4 [5.9–18.4]	6.5 [4.3–9.6]	6.1 [3.5–9.5]
Timor Leste	Household contacts	11.9 [8.6–16.5]	17.2 [11.0–33.6]	8.6 [5.7–12.3]	7.5 [2.0–13.3]
	30% coverage of low-BMI population	12.4 [8.6–18.1]	22.2 [14.3–40.2]	11.8 [8.3–16.9]	13.2 [4.4–20.4]

**Table 3:** Summary of mortality impact in each country in the SE Asia Region by nutritional support.

Deaths averted (2023–2030)	Strategy	Status quo comparator		Improved TB cascade comparator	
		Pct reduction in cumulative deaths (2023–2030)	Incremental deaths reductions in 2030 relative to 2015 attributable to nutritional interventions	Pct reduction in cumulative deaths (2023–2030)	Incremental deaths reductions in 2030 relative to 2015 attributable to nutritional interventions
Bangladesh	Household contacts	4.9 [3.8–6.3]	6.5 [4.6–12.0]	3.5 [2.2–4.8]	2.6 [1.2–4.2]
	30% coverage of low-BMI population	25.1 [20.9–29.9]	34.9 [25.9–60.1]	23.7 [19.4–28.0]	16.8 [9.4–25.3]
Bhutan	Household contacts	0.33 [0.07–1.6]	0.35 [0.06–1.7]	0.2 [0.03–0.9]	0.09 [0.01–0.5]
	30% coverage of low-BMI population	11.6 [8.2–17.1]	13.7 [9.4–19.9]	11.4 [8.1–16.5]	5.3 [3.2–8.5]
DPR Korea	Household contacts	9.0 [5.5–15.3]	12.8 [7.4–24.2]	7.1 [2.9–11.6]	4.6 [0.7–10.4]
	30% coverage of low-BMI population	11.4 [6.4–18.7]	18.2 [10.3–33.0]	11.1 [6.0–17.7]	7.8 [1.4–15.2]
India	Household contacts	4.8 [3.6–6.2]	6.2 [4.5–11.2]	3.5 [2.0–4.6]	2.4 [1.0–3.8]
	30% coverage of low-BMI population	26.0 [22.4–30.8]	35.5 [28.6–58.4]	24.6 [21.5–28.1]	17.1 [10.2–25.1]
Indonesia	Household contacts	9.0 [6.9–11.7]	16.4 [10.0–31.9]	5.4 [2.9–7.7]	3.3 [1.1–7.6]
	30% coverage of low-BMI population	19.2 [15.0–24.0]	38.7 [25.4–70.7]	17.2 [13.5–21.7]	11.3 [4.9–20.9]
Myanmar	Household contacts	7.9 [6.4–11.5]	11.1 [8.3–26.0]	6.1 [4.4–9.1]	4.5 [2.7–11.4]
	30% coverage of low-BMI population	16.8 [13.3–21.5]	25.6 [19.3–50.1]	16.8 [13.3–20.9]	12.4 [8.3–24.7]
Nepal	Household contacts	3.3 [2.4–4.2]	4.3 [2.8–7.0]	2.4 [1.4–3.2]	1.3 [0.4–2.7]
	30% coverage of low-BMI population	18.9 [13.7–23.4]	29.0 [20.4–44.2]	18.9 [14.7–22.3]	11.4 [5.7–23.6]
Sri Lanka	Household contacts	0.5 [0.3–0.7]	0.53 [0.33–0.85]	0.3 [0.1–0.6]	0.2 [0.07–0.54]
	30% coverage of low-BMI population	18.2 [14.3–21.9]	20.9 [16.4–28.2]	18.5 [15.0–22.3]	12.6 [9.4–19.4]
Thailand	Household contacts	2.3 [1.5–3.3]	2.8 [1.8–4.6]	1.8 [0.8–2.9]	1.2 [0.4–2.1]
	30% coverage of low-BMI population	13.7 [10.0–19.0]	17.7 [11.6–27.6]	13.3 [9.8–17.9]	9.3 [5.4–14.1]
Timor Leste	Household contacts	12.9 [9.7–16.6]	19.5 [14.2–35.8]	9.5 [5.6–13.4]	6.2 [1.3–13.7]
	30% coverage of low-BMI population	21.2 [16.2–26.8]	36.2 [27.1–53.9]	20.8 [15.6–26.3]	15.4 [3.8–27.1]

Nutritional interventions will thus play an important role in efforts aimed at ending TB in the Region, and should be addressed as a priority. However, to achieve the greatest possible reductions in TB incidence and mortality, there is also a need for a comprehensive approach including improvements in the quality and coverage of TB services, as well as broader preventive strategies. In particular, the population attributable fraction for undernutrition accounts for around 50% in the Region.^2^ Although a large fraction, this leaves half of TB to be addressed by other means. Addressing a range of risk factors for TB would maximise the impact of determinant-led prevention measures. For example, smoking cessation has been shown to reduce TB transmissibility,^22^ as well as reversing most smoking-related immunodeficiency within weeks.^23^ Diabetes is known to exacerbate outcomes at all stages of TB, from the risk of developing disease to recurrence^24^; effective glycaemic control could likewise have important effects on TB incidence and mortality at the population level. As well as measures to address undernutrition, therefore, efforts to improve broader determinants of population health will have important contributions towards meeting the End TB goals.

In future, effective TB vaccines would play an equally important role in population prevention.^8^ However, such vaccines cannot be a substitute for the preventive strategies discussed above. They are likely to be only partially effective, for example with early trials of the M72/AS01e vaccine suggesting only around 50% efficacy in preventing disease amongst adults and adolescents with evidence of TB infection.^25^ Moreover, undernutrition may compromise TB vaccine efficacy as it does for other vaccines,^26^^,^^27^ and likewise for other immunomodulating risk factors.^28^ Thus, future TB vaccines should be seen as one important arm of a broadly preventive strategy, that simultaneously aims to address the key determinants of TB.

In any such broadly preventive strategy, the need for a multisectoral, multidisciplinary approach cannot be overemphasised. As well as offering nutritional support, there is a critical need to ensure uptake of these services. Doing so will require engagement with all sectors relevant for nutrition support and specifically catering to vulnerable populations. This would mean going beyond just the health sector, a strategic approach that is emphasised in the South-East Asia Regional Strategic Plan towards ending TB, 2021–2025.^29^ To bring these sectors together, the highest possible political level commitment providing oversight to such coordination measures would be important.^30^ Creation of such platforms has already been emphasised in declarations from several high-level Regional meetings in 2017, 2018, 2021 and more recently in 2023 that led to the Gandhinagar Declaration.^30^, ^31^, ^32^ International agencies such as the IOM and WFP could also play an important role in collaborating with WHO to develop a combined strategy for addressing undernutrition in general population as well as vulnerable populations. Such international support would be even more important in countries facing geopolitical challenges that hinder access and availability of care. Addressing social determinants needs strong grassroots involvement. In particular, such ambitions cannot be achieved without a strong engagement of the community in planning, monitoring and delivery, to make the services acceptable and accessible.

As with any modelling, our analysis has limitations to note. In modelling the potential impact of addressing undernutrition in the general population, we have dichotomised undernutrition as being associated with BMI <18.5, whereas in reality the association between BMI and TB operates on a continuous scale.^6^^,^^7^ Thus, nutritional supplementation in the general population may benefit even those with BMI above 18.5: by ignoring such effects, our analysis is likely to be conservative with respect to the potential impact of nutritional supplementation, on TB burden. On the other hand, BMI-based thresholds will be critical from an implementation perspective, as they will provide clear, easily-measured criteria for eligibility for nutritional support. This leads to an important question for future work: what would be the optimal choice of BMI threshold, for such population-level measures in a given setting? Addressing this question will require not just epidemiological impact (e.g. expanding studies like the RATIONS trial to other BMI groups), but also cost implications of more or less stringent criteria.

Relatedly, our analysis does not address cost implications of the interventions we modelled, whether from the programmatic or patient perspective. Recent work using Markov state transition models showed that nutritional support amongst contacts would be cost-effective.^11^ In future, it would be valuable to extend this analysis to population-level interventions (i.e. beyond contacts), in addition to incorporating transmission-reducing impact such as those we have modelled here.

Certain simplifications bear mention, in our modelling of associations between undernutrition and TB. In particular, we have captured the threefold relative risk of TB amongst people with undernutrition, compared to those with normal BMI and above, assuming that this relative risk arises from an increased risk of progression from TB infection to active disease (the factor k in Fig. 1a). In reality, it is likely that those with undernutrition also face an increased risk of exposure to TB. For example, those living in urban slums are more likely to have undernutrition, while also living in a higher-transmission environment, than the general population. Further work to incorporate these factors would benefit from systematic data on, for example, the relative risk of TB infection (as well as TB disease) amongst those with undernutrition, compared to those in the general population.

Amongst other limitations, while the RATIONS study population was a largely rural community in Jharkhand, India, with high levels of multidimensional poverty and thus the effects of the intervention may not be generalisable to patients with lower prevalence and severity of undernutrition. It should be noted that TB patients in this population were similar in nutritional status to patients in other states such as Madhya Pradesh^33^ and Chhattisgarh,^34^ as well as those managed by India's national tuberculosis programme.^12^^,^^35^ Nonetheless further studies of nutritional support elsewhere in the country will be invaluable in validating our projections at the national level. Relatedly, by modelling each country in the Region at the national level, we have not addressed subnational variation in the prevalence of undernutrition. In large and heterogeneous countries such as Bangladesh, India and Indonesia in particular, in the immediate term there may be a need to focus interventions in those regions/provinces and populations where undernutrition is highest. While we have modelled the adverse impact of COVID-19 on routine TB services in the Region, it is also possible that the economic shock from the pandemic also exacerbated existing levels of undernutrition: our model does not incorporate such effects. Our model entails several other simplifications including, for example, the lack of age structure. We have used data for the prevalence of undernutrition in adults, but its prevalence is typically higher in children.^36^^,^^37^ By ignoring these differences, our model projections may therefore underestimate the impact of nutritional interventions. On the other hand, the prevalence of TB infection is lower in children, which would tend to bias model projections in the opposite direction. Overall, addressing these simplifications would be an important area for future, more refined modelling. Additionally, the impact of nutritional interventions on TB may vary with age, for example because of their effects on child and adolescent development, and further work is needed to inform how these effects might vary. Finally, we have focused here on the effect of nutritional status on the risk of developing TB, whereas there is evidence to suggest that nutritional support can also improve survival and treatment outcomes amongst those already suffering TB disease.^38^^,^^39^ Although such benefits are outside the scope of our current analysis, they mean that our results for TB mortality reductions are likely underestimates.

In conclusion, although enhancing TB-focused services will undoubtedly be important in the global TB response, there is a limit to what these measures can achieve in the absence of prevention, particularly in the wake of COVID-related disruptions. In the SE Asia Region, the prominence of undernutrition as a driver for TB burden makes it a natural target for interventions aimed at preventing TB. Addressing this, and other determinants of TB, will become increasingly critical in the drive to End TB.

## Contributors

VB, SR conceptualized the study; SM and NA developed the modelling approach; and SM performed the modelling. AB reviewed the modelling results and provided inputs for further refining the model. SM and NA wrote a first draft of the manuscript. All authors analysed and interpreted the results, contributed to the final draft and approved the version for submission to the journal.

## Data sharing statement

The study employed publicly available data, which are cited in this paper. Simulation codes are also available upon request from the corresponding author.

## Declaration of interests

SM worked in the capacity of an independent consultant. VB, SR are staff of the WHO South-East Asia Regional Office. AB is supported by Yenepoya Medical College. NA is staff of WHO Geneva, and is affiliated with Imperial College London, where he earlier received support from the UK Medical Research Council and the Bill & Melinda Gates Foundation.
