# Supplementary material for: The potential impact on tuberculosis of interventions to reduce undernutrition in the WHO South-East Asian Region: a modelling analysis
Source: Lancet Reg Health Southeast Asia. 2024 May 16;31:100423. doi: 10.1016/j.lansea.2024.100423 (PMC11827067; doi:10.1016/j.lansea.2024.100423)
Supplement: Supplementary Figure and Tables [file mmc1.docx]

**Supplementary information**

**The potential impact on tuberculosis of interventions to reduce undernutrition in the WHO South-East Asian Region: a modelling analysis**

Sandip Mandal^1^, Vineet Bhatia^2^, Anurag Bhargava^3^, Suman Rijal^2^, Nimalan Arinaminpathy^4^

^1^ John Snow India, New Delhi, India

^2^ World Health Organization, South-East Asia Regional Office, New Delhi, India

^3^ Department of General Medicine, Yenepoya Medical College, Karnataka, India

^4^ MRC Centre for Global Infectious Disease Analysis, Imperial College London, London, UK

Table of Contents

[1. Model structure 2](#_Toc160360745)

[2. Equations and parameter tables 2](#_Toc160360746)

[3. Model calibration 16](#_Toc160360747)

**4.**  **Posterior distributions of calibrated parameters…………………………………………………..20**

[5. Modelling interventions 25](#_Toc160360748)

[6. Sensitivity analyses 30](#_Toc160360749)

[7. References 30](#_Toc160360750)

# Model structure

Figure 1 gives a schematic illustration of the overall model structure, with all model parameters listed in Table S1. In brief, panel (A) shows the population divided into three nutritional strata: those with normal BMI (left-hand column); with low BMI (right-hand column); and those who had low BMI, but have received nutritional rehabilitation (middle column). We assume that each column carries its own risks of progression from TB infection to active disease, as determined by the relative risk of TB amongst those with low BMI, and the effectiveness of nutritional rehabilitation in reducing this risk. For the latter, we draw from a recent study^1^. Panel (B) shows a continuation of panel A, with emphasis on the care cascade once individuals develop TB.

***
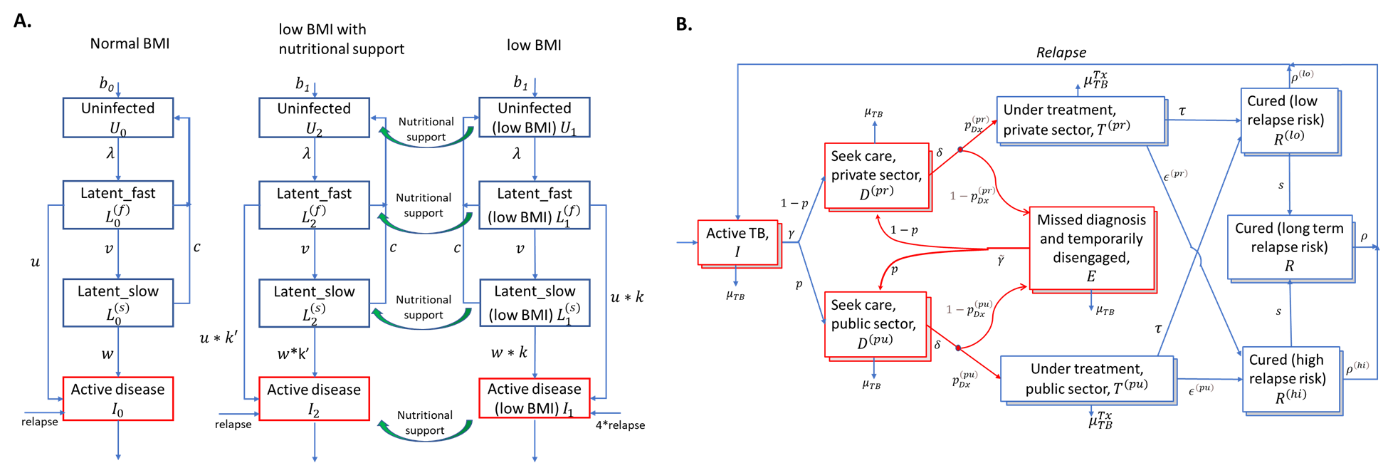
***

**Figure S1. Schematic illustration of the model structure.** Parameters are defined below, and in Table 1. (main text) Infectious compartments contributing the force-of-infection are shown in red. For clarity, the diagram omits certain rates incorporated in the model, including: self-cure; exogenous reinfection; and background mortality.

(A) Rates of progression from LTBI to active TB, low BMI relative to normal BMI: estimated to fit with the relative risk of TB among malnutrition (~3.1). (B) Each compartment consists of two layers: one with normal BMI and the other with low BMI. Among the latter, relapse rate is 4 times and TB-mortality is 2 times higher than the normal BMI population.

# Equations and parameter tables

Governing equations of the model are as follows. All state variables are written as proportions of the population (not as absolute numbers). Depending on the BMI-status each state variable has three categories represented by the subscript index $i$, where $i=0$ denotes ‘normal BMI’, $i$= 1 denotes ‘low BMI’ and $i$ = 2 denotes ‘low BMI with rehabilitation’.

In all equations below, the term $k_{i}$ represents a nutrition-dependent modifier on rates relating to the development of active TB amongst those with TB infection (with $k_{0}=1,$ and $k_{1}>k_{2}>1$). Values of these and other parameters are given in Table 1. Similarly, $n_{i}$ is a nutrition-dependent modifier on rates relating to TB mortality, while $m_{i}$ is a nutrition-dependent modifier on rates relating to post-treatment, endogenous relapse.

Uninfected ($U$):

$$\frac{dU_{i}}{dt}=b+c(L_{i}^{f}+L_{i}^{s})-(\lambda+\mu)U_{i}$$

for a birth-rate $b$; force-of-infection $\lambda$; rate of clearance of LTBI $c$; and background mortality rate $\mu$.

Latent, ‘fast’ infection ($L^{f}$):

$$\frac{dL_{i}^{f}}{dt}=\lambda U_{i}+\left( 1-h \right)\lambda(R_{i}^{(hi)}+R_{i}^{(lo)}+R_{i})-\left( \mu+k_{i}u+v+c \right)L_{i}^{f}$$

for a progression rate $(k_{i}u)$; a ‘stabilisation’ rate (to latent ‘slow’ status) $v$; and protection from reinfection $h$, amongst those previously infected. The parameter $k_{0}=1$ for ‘normal BMI’ and have higher values (estimated, see table 1) for those with ‘low BMI’ ($k_{1}$and $k_{2})$.

Latent, ‘slow’ infection ($L^{s}$):

$$\frac{dL_{i}^{s}}{dt}=vL_{i}^{f}-\left( \mu+c+k_{i}w \right)L_{i}^{s}$$

for a reactivation rate ${(k}_{i}w)$.

Active TB ($I$):

$$\frac{dI_{i}}{dt}=k_{i}u L_{i}^{f}+k_{i}w L_{i}^{s}+{m_{i}\rho}_{hi}R_{i}^{(hi)}+m_{i}\rho_{lo}R_{i}^{(lo)}+\rho R_{i}-(\gamma+{n_{i}\mu}_{TB}+\sigma)I_{i}$$

for relapse rates $\rho^{\left( hi \right)}, \rho^{\left( lo \right)}, \rho$; careseeking rate $\gamma$; TB mortality rate ${n_{i} \mu}_{TB}$; and self-cure rate $\sigma$. $m_{i}$is relative relapse risk for the ‘low-BMI’ population and its value for ‘normal BMI’ $m_{0}=1$, $m_{2}=1$ and $m_{1}=4$. TB mortality is two times higher for those with ‘low-BMI’^2^. Therefore, $n_{0}=1$ , $n_{2}=1$ and $n_{1}=2$.

Here, $p$ and $(1-p)$ are, respectively, the proportion-of-presentation to healthcare providers in the public and private sectors. The factor $\gamma$ represents the rate at which individuals present for diagnosis: it is assumed to increase in time, motivated by data showing a steady increase in the presumptive rate for TB in India, as well as to capture the decline in incidence in WHO estimates^3^.

Presented for diagnosis with provider type $s$ ($D^{\left( s \right)}$):

$$\frac{dD_{i}^{(pu)}}{dt}= p (\gamma I_{i}+ \tilde{\gamma} E_{i})-\left( {n_{i} \mu}_{TB}+\sigma+\delta\right)D_{i}^{(pu)}$$

$$\frac{dD_{i}^{(pr)}}{dt}=(1-p) (\gamma I_{i}+ \tilde{\gamma} E_{i})-\left( {n_{i} \mu}_{TB}+\sigma+\delta\right)D_{i}^{(pr)}$$

Here,  $\tilde{\gamma}$ is analogous to$\gamma$, but attached to individuals who remain undiagnosed despite having previously sought care (i.e. compartment $E,$below).

On TB treatment with provider type $s$ ($T^{\left( s \right)}$):

$$\frac{dT_{i}^{(pu)}}{dt}=\delta p_{Dx}^{\left( pu \right)}D_{i}^{(pu)}-\left( \mu_{TB}^{Tx}+\tau+\epsilon^{\left( pu \right)} \right)T_{i}^{(pu)}$$

$$\frac{dT_{i}^{(pr)}}{dt}=\delta p_{Dx}^{\left( pr \right)}D_{i}^{(pr)}-\left( \mu_{TB}^{Tx}+\tau+\epsilon^{\left( pr \right)} \right)T_{i}^{(pr)}$$

for a treatment completion rate $\tau$; and a treatment interruption rate $\epsilon^{\left( s \right)}$.

Missed diagnosis and temporarily disengaged from careseeking ($E$):

$$\frac{dE_{i}}{dt}=\delta\left( 1-p_{Dx}^{\left( pu \right)} \right)D_{i}^{(pu)}+\delta\left( 1-p_{Dx}^{\left( pr \right)} \right)D_{i}^{(pr)}-\left( {n_{i} \mu}_{TB}+\sigma+\tilde{\gamma} \right)E_{i}$$

Recovered with low relapse risk, following treatment completion ($R^{\left( lo \right)}$):

$$\frac{dR_{i}^{(lo)}}{dt}=\tau\left( T_{i}^{(pu)}+T_{i}^{(pr)} \right)-[(1-h)\lambda+{m_{i}\rho}^{\left( lo \right)}+\mu+s]R_{i}^{(lo)}$$

for a rate of ‘stabilisation’ of relapse risk $s$.

Recovered with high relapse risk, following treatment non-completion or self-cure ($R^{\left( hi \right)}$):

$$\frac{dR_{i}^{(hi)}}{dt}=\left( \epsilon^{\left( pu \right)}T_{i}^{(pu)}+\epsilon^{\left( pr \right)}T_{i}^{(pr)} \right)-\left[ \left( 1-h \right)\lambda+{m_{i}\rho}^{\left( hi \right)}+\mu+s \right]R_{i}^{\left( hi \right)}+\sigma\left( I_{i}+E_{i}+D_{i}^{(pu)}+D_{i}^{(pr)} \right)$$

Long-term, ‘stabilised’ relapse risk ($R$):

$$\frac{dR_{i}}{dt}=s\left( R_{i}^{(hi)}+R_{i}^{(lo)} \right)-[(1-h)\lambda+m_{i}\rho+\mu]R_{i}$$

Force-of-infection $(\lambda)$:

$$\lambda=\beta(I_{i}+E_{i}+D_{i}^{(pu)}+D_{i}^{(pr)})$$

for a rate-of-transmission $\beta$.

*Table S1: Table of parameters (As an example, calibrated values shown for India)*

| Parameter | | Symbol | Value | Source/Notes |
| --- | --- | --- | --- | --- |
| Natural history | | | | |
| **Infection rate (average number of annual infections per case)** | | $\beta$ | 19 (8 – 29) | Model calibration, with priors U[0, 30]. Estimated values vary across the countries. |
| **Per-capita annual rate of progression from ‘fast’ latent infection (population with normal BMI)** | | $u$ | 0.10 (0.01 – 0.24) | Calibration: Menzies (2018)^4^ for central value, and U[0.1 - 20] on multiplying factor |
| **Per-capita annual rate of stabilisation from ‘fast’ to ‘slow’ latent status** | | $v$ | 0.88  (0.66 – 0.95) | Menzies (2018)^4^ for central value, and taking uniform priors of +/- 25% |
| **Per-capita annual rate of reactivation from ‘slow’ latent infection (population with normal BMI)** | | $w$ | 0.0018  (0.0003 – 1.01) | Calibration: Menzies (2018)^4^ for central value, and U[0.1 - 20] on multiplying factor |
| **Relative per-capita annual progression and reactivation rate for low-BMI population** | | $k_{1}$ | 1.76 (1.66 – 1.86) | Calibration: relative risk of TB among lo-BMI relative to normal-BMI is 3.2 [3.1 – 3.3]^5^ |
| **Reduction in per-capita rates of progression and reactivation amongst those with undernutrition, as a result of nutritional support** | | $y,$  where  $k_{2}=\left( 1-y \right)k_{1}$ | 0.29 | Chosen to yield same incidence reductions as observed amongst undernourished household contacts in Bhargava et al (2023)^6^. |
| **Per-capita annual rate of self-clearance of TB infection** | | $c$ | 0.027  (0.022 – 0.035) | Emery (2021)^7^ for central value, and taking uniform priors of +/- 25% |
| **Per-capita annual rate of TB mortality while untreated** | | $\mu_{TB}$ | 0.33 (0.11 – 0.54)  (for normal-BMI and for low-BMI with nutritional support)  ${2*\mu}_{TB}$  (for low-BMI)^2^ | Calibration:  Value for normal BMI  calibrated to meet WHO estimated TB mortality.  Value for low BMI chosen to represent higher risk of mortality amongst those with undernutrition.   Value for low BMI with nutritional support chosen to match that for normal BMI, consistent with treatment outcome findings in^1^, and assuming these to apply to mortality while untreated as well. |
| **Per-capita annual rate of TB mortality while in treatment** | | $\mu_{TB}^{Tx}$ | 0.098  (for normal-BMI and for low-BMI with nutritional support)  0.15  (for low-BMI) | Corresponding to 4% CFR during treatment among normal-BMI^8^  and 6% for low BMI population.  Value for low BMI with nutritional support chosen to match that for normal BMI, consistent with treatment outcome findings in Bhargava et al (2023)^1^. |
| **Per-capita annual rate of TB self-cure** | | $\sigma$ | 0.55 (0.03 – 1.6) | Tiemersma et al., (2011)^9^ for central value, and U[0.1 - 10] on multiplying factor |
| **Protection from reinfection amongst those with prior infection** | | $h$ | 0.41 (0.13 – 0.72) | Andrews (2012)^10^ [4], assuming uniform priors of +/-25% |
| **Per-capita annual rate of relapse in first two years after treatment completion** | | $\rho^{\left( lo \right)}$ | 0.032  (0.024 – 0.04)  (for normal-BMI) | Thomas A et al (2005)^11^, Romanowski (2019)^12^, Menzies (2009)^13^ and Weis (1994)^14^, with uniform prior using intervals of ± 5%. For low-BMI relapse rate is 4 times higher than normal-BMI |
| **Per-capita annual rate of relapse in first two years after self-cure or incomplete treatment** | | $\rho^{\left( hi \right)}$ | 0.14  (0.11 – 0.17)  (for normal-BMI) |  |
| **Per-capita annual rate of relapse >two years after last TB episode** | | $\rho$ | 0.0015  (0.0011 – 0.0018)  (for normal-BMI) | Most relapse occurs in first two years after recovery: Guerra-Assuncao (2015)^15^ |
| **Reduction in per-capita rates of post-treatment relapse amongst those with undernutrition, as a result of nutritional support** | | $z,$  where  $m_{2}=\left( 1-z \right)m_{1}$ | 0.75 | Consistent with study findings that nutritional support achieves 5% increase in bodyweight amongst approximately 50% of patients by 2 months^1^, and that this weight gain is associated with approximately 50% reduction in post-treatment relapse risk^16^. |
| **Per-capita annual rate of ‘stabilising’ from high to low relapse risk** | | $s$ | 0.5 |  |
| TB services | | | | |
| **Rate-of-presentation to care, first careseeking visit** | | $\gamma$ | 7.5  (2.3 – 9.8) | Model calibration, with priors U[0.1, 10] |
| **Rate-of-presentation to care, second and subsequent careseeking visits** | | $\tilde{\gamma}$ | 14.8  (3.2 – 23.6) | Model calibration, taking U[1, 24] |
| **Probability that a TB patient visits public provider, per careseeking attempt** | | $p$ | 0.56  (0.26 – 0.72) | Model calibration |
| **Per-capita annual rate of offering diagnosis** | | $\delta$ | 52 | Assumption, corresponding to 1 week |
| **Probability of successful TB diagnosis and treatment initiation per careseeking visit** | Public sector | $p_{Dx}^{\left( pu \right)}$ | 0.82  (0.75 – 0.89) | U[0.3, 0.9], motivated by Subbaraman (2016)^17^ |
|  | Private sector | $p_{Dx}^{\left( pr \right)}$ | 0.20  (0.10 – 0.30) | U[0.1, 0.4], assumption |
| **Per-capita annual rate of treatment completion** | | $\tau$ | 2 | Corresponds to average duration of 6 months |
| **Per-capita annual rate of treatment interruption** | Public sector | $\epsilon^{\left( pu \right)}$ | 0.32  (0.12 – 0.63) | Calculated using $\epsilon^{\left( pu \right)}=\frac{1-P}{P}\tau$, for treatment completion rate $P$, and assuming U[0.75, 0.95] for $P$ |
|  | Private sector | $\epsilon^{\left( pr \right)}$ | 1.4  (0.5 – 2.8) | As above, but assuming U[0.4, 0.8] for $P$ |
| Demographics | | | | |
| **Per-capita annual rate of background mortality** | | $\mu$ | 1/67 | Corresponds to average lifespan of 67 years (World Bank 2021)^18^ |

**Table S1. List of model parameters, values and sources.** The notation $U[x,y]$ denotes a uniform probability distribution on the range $[x,y]$. Otherwise, parameter values in round brackets show 95% percentiles from the respective marginal posterior densities.

*Table S2: Table of parameters (calibrated values shown for all other countries except India). The description of each symbol and other details are the same as in Table S1.*

| Country: Bangladesh | | |
| --- | --- | --- |
| Parameter Symbol | Value | |
| $\beta$ | 20 (9 – 29) | |
| $u$ | 0.11 (0.02 – 0.23) | |
| $v$ | 0.89 (0.66 – 1.08) | |
| $w$ | 0.0017 (0.0002 – 0.0043) | |
| $k_{1}$ | 1.70 (1.56 – 1.88) | |
| $y$ | 0.285 | |
| $c$ | 0.028 (0.021 – 0.035) | |
| $\mu_{TB}$ | 0.22 (0.07 – 0.50) (for normal-BMI) | ${2*\mu}_{TB}$(for low-BMI)^2^ |
| $\mu_{TB}^{Tx}$ | 0.098 (for normal-BMI) | 0.15 (for low-BMI) |
| $\sigma$ | 0.64 (0.05 – 1.6) | |
| $h$ | 0.42 (0.12 – 0.74) | |
| $\rho^{\left( lo \right)}$ | 0.031 (0.024 – 0.04) (for normal-BMI) | |
| $\rho^{\left( hi \right)}$ | 0.14 (0.11 – 0.17) (for normal-BMI) | |
| $\rho$ | 0.0015 (0.0011 – 0.0019) (for normal-BMI) | |
| $z$ | 0.75 | |
| $s$ | 0.5 | |
| $\gamma$ | 7.6 (3.1 – 9.9) | |
| $\tilde{\gamma}$ | 13.7 (4.0 – 23.4) | |
| $p$ | 0.54 (0.29 – 0.72) | |
| $\delta$ | 52 | |
| $p_{Dx}^{\left( pu \right)}$ | 0.83 (0.75 – 0.89) | |
| $p_{Dx}^{\left( pr \right)}$ | 0.19 (0.10 – 0.29) | |
| $\tau$ | 2 | |
| $\epsilon^{\left( pu \right)}$ | 0.36 (0.12 – 0.65) | |
| $\epsilon^{\left( pr \right)}$ | 1.3 (0.5 – 2.8) | |
| $\mu$ | 1/72 | |

| Country: Bhutan | | |
| --- | --- | --- |
| Parameter Symbol | Value | |
| $\beta$ | 6.5 (3.2 – 10.5) | |
| $u$ | 0.56 (0.30 – 0.86) | |
| $v$ | 0.92 (0.68 – 1.08) | |
| $w$ | 0.0007 (0.0001 – 0.0021) | |
| $k_{1}$ | 4.45 (2.01 – 9.76) | |
| $y$ | 0.34 | |
| $c$ | 0.025 (0.021 – 0.032) | |
| $\mu_{TB}$ | 0.34 (0.15 – 0.66) (for normal-BMI) | ${2*\mu}_{TB}$(for low-BMI)^2^ |
| $\mu_{TB}^{Tx}$ | 0.098 (for normal-BMI) | 0.15 (for low-BMI) |
| $\sigma$ | 0.26 (0.03 – 0.92) | |
| $h$ | 0.65 (0.39 – 0.74) | |
| $\rho^{\left( lo \right)}$ | 0.032 (0.024 – 0.04) (for normal-BMI) | |
| $\rho^{\left( hi \right)}$ | 0.14 (0.11 – 0.17) (for normal-BMI) | |
| $\rho$ | 0.0013 (0.0011 – 0.0017) (for normal-BMI) | |
| $z$ | 0.75 | |
| $s$ | 0.5 | |
| $\gamma$ | 8.3 (5.2 – 9.9) | |
| $\tilde{\gamma}$ | 10.9 (3.2 – 22.9) | |
| $p$ | 0.54 (0.37 – 0.69) | |
| $\delta$ | 52 | |
| $p_{Dx}^{\left( pu \right)}$ | 0.84 (0.75 – 0.89) | |
| $p_{Dx}^{\left( pr \right)}$ | 0.15 (0.10 – 0.25) | |
| $\tau$ | 2 | |
| $\epsilon^{\left( pu \right)}$ | 0.32 (0.11 – 0.65) | |
| $\epsilon^{\left( pr \right)}$ | 1.5 (0.54 – 2.86) | |
| $\mu$ | 1/72 | |

| Country: DPR Korea | | |
| --- | --- | --- |
| Parameter Symbol | Value | |
| $\beta$ | 25 (13 – 29) | |
| $u$ | 0.04 (0.01 – 0.11) | |
| $v$ | 0.88 (0.67 – 1.08) | |
| $w$ | 0.0037 (0.0004 – 0.007) | |
| $k_{1}$ | 1.86 (1.75 – 1.98) | |
| $y$ | 0.31 | |
| $c$ | 0.026 (0.021 – 0.034) | |
| $\mu_{TB}$ | 0.17 (0.07 – 0.32) (for normal-BMI) | ${2*\mu}_{TB}$(for low-BMI)^2^ |
| $\mu_{TB}^{Tx}$ | 0.098 (for normal-BMI) | 0.15 (for low-BMI) |
| $\sigma$ | 0.15 (0.02 – 0.51) | |
| $h$ | 0.50 (0.13 – 0.74) | |
| $\rho^{\left( lo \right)}$ | 0.032 (0.024 – 0.04) (for normal-BMI) | |
| $\rho^{\left( hi \right)}$ | 0.14 (0.11 – 0.17) (for normal-BMI) | |
| $\rho$ | 0.0015 (0.0011 – 0.0019) (for normal-BMI) | |
| $z$ | 0.75 | |
| $s$ | 0.5 | |
| $\gamma$ | 3.7 (1.1 – 9.3) | |
| $\tilde{\gamma}$ | 3.3 (1.1 – 17.8) | |
| $p$ | 0.49 (0.28 – 0.68) | |
| $\delta$ | 52 | |
| $p_{Dx}^{\left( pu \right)}$ | 0.82 (0.75 – 0.89) | |
| $p_{Dx}^{\left( pr \right)}$ | 0.17 (0.10 – 0.29) | |
| $\tau$ | 2 | |
| $\epsilon^{\left( pu \right)}$ | 0.34 (0.12 – 0.64) | |
| $\epsilon^{\left( pr \right)}$ | 1.3 (0.5 – 2.9) | |
| $\mu$ | 1/73 | |

| Country: Indonesia | | |
| --- | --- | --- |
| Parameter Symbol | Value | |
| $\beta$ | 19 (6 – 29) | |
| $u$ | 0.16 (0.03 – 0.28) | |
| $v$ | 0.91 (0.68 – 1.08) | |
| $w$ | 0.0008 (0.0001 – 0.0039) | |
| $k_{1}$ | 1.81 (1.68 – 1.98) | |
| $y$ | 0.29 | |
| $c$ | 0.024 (0.021 – 0.031) | |
| $\mu_{TB}$ | 0.22 (0.09 – 0.37) (for normal-BMI) | ${2*\mu}_{TB}$(for low-BMI)^2^ |
| $\mu_{TB}^{Tx}$ | 0.098 (for normal-BMI) | 0.15 (for low-BMI) |
| $\sigma$ | 0.57 (0.04 – 1.5) | |
| $h$ | 0.56 (0.15 – 0.74) | |
| $\rho^{\left( lo \right)}$ | 0.033 (0.024 – 0.039) (for normal-BMI) | |
| $\rho^{\left( hi \right)}$ | 0.15 (0.11 – 0.17) (for normal-BMI) | |
| $\rho$ | 0.0014 (0.0011 – 0.0018) (for normal-BMI) | |
| $z$ | 0.75 | |
| $s$ | 0.5 | |
| $\gamma$ | 6.4 (2.9 – 9.7) | |
| $\tilde{\gamma}$ | 7.9 (2.6 – 22.7) | |
| $p$ | 0.45 (0.26 – 0.69) | |
| $\delta$ | 52 | |
| $p_{Dx}^{\left( pu \right)}$ | 0.82 (0.75 – 0.89) | |
| $p_{Dx}^{\left( pr \right)}$ | 0.18 (0.10 – 0.29) | |
| $\tau$ | 2 | |
| $\epsilon^{\left( pu \right)}$ | 0.34 (0.11 – 0.64) | |
| $\epsilon^{\left( pr \right)}$ | 1.6 (0.6 – 2.9) | |
| $\mu$ | 1/69 | |

| Country: Myanmar | | |
| --- | --- | --- |
| Parameter Symbol | Value | |
| $\beta$ | 21 (11 – 29) | |
| $u$ | 0.05 (0.01 – 0.16) | |
| $v$ | 0.99 (0.67 – 1.08) | |
| $w$ | 0.0039 (0.0006 – 0.0064) | |
| $k_{1}$ | 1.79 (1.65 – 2.22) | |
| $y$ | 0.30 | |
| $c$ | 0.032 (0.021 – 0.035) | |
| $\mu_{TB}$ | 0.18 (0.05 – 0.73) (for normal-BMI) | ${2*\mu}_{TB}$(for low-BMI)^2^ |
| $\mu_{TB}^{Tx}$ | 0.098 (for normal-BMI) | 0.15 (for low-BMI) |
| $\sigma$ | 0.38 (0.02 – 1.2) | |
| $h$ | 0.47 (0.12 – 0.73) | |
| $\rho^{\left( lo \right)}$ | 0.034 (0.024 – 0.04) (for normal-BMI) | |
| $\rho^{\left( hi \right)}$ | 0.16 (0.11 – 0.17) (for normal-BMI) | |
| $\rho$ | 0.0016 (0.0012 – 0.0019) (for normal-BMI) | |
| $z$ | 0.75 | |
| $s$ | 0.5 | |
| $\gamma$ | 7.0 (1.7 – 9.9) | |
| $\tilde{\gamma}$ | 2.8 (1.1 – 14.7) | |
| $p$ | 0.6 (0.33 – 0.73) | |
| $\delta$ | 52 | |
| $p_{Dx}^{\left( pu \right)}$ | 0.86 (0.76 – 0.89) | |
| $p_{Dx}^{\left( pr \right)}$ | 0.25 (0.11 – 0.30) | |
| $\tau$ | 2 | |
| $\epsilon^{\left( pu \right)}$ | 0.23 (0.11 – 0.56) | |
| $\epsilon^{\left( pr \right)}$ | 0.97 (0.5 – 2.8) | |
| $\mu$ | 1/67 | |

| Country: Nepal | | |
| --- | --- | --- |
| Parameter Symbol | Value | |
| $\beta$ | 24 (14 – 29) | |
| $u$ | 0.09 (0.01 – 0.18) | |
| $v$ | 0.90 (0.68 – 1.08) | |
| $w$ | 0.0017 (0.0002 – 0.0047) | |
| $k_{1}$ | 1.96 (1.74 – 2.29) | |
| $y$ | 0.29 | |
| $c$ | 0.026 (0.021 – 0.034) | |
| $\mu_{TB}$ | 0.7 (0.27 – 1.4) (for normal-BMI) | ${2*\mu}_{TB}$(for low-BMI)^2^ |
| $\mu_{TB}^{Tx}$ | 0.098 (for normal-BMI) | 0.15 (for low-BMI) |
| $\sigma$ | 0.76 (0.06 – 1.59) | |
| $h$ | 0.39 (0.11 – 0.73) | |
| $\rho^{\left( lo \right)}$ | 0.032 (0.024 – 0.04) (for normal-BMI) | |
| $\rho^{\left( hi \right)}$ | 0.14 (0.11 – 0.17) (for normal-BMI) | |
| $\rho$ | 0.0015 (0.0011 – 0.0019) (for normal-BMI) | |
| $z$ | 0.75 | |
| $s$ | 0.5 | |
| $\gamma$ | 6.1 (1.9 – 9.8) | |
| $\tilde{\gamma}$ | 11.5 (1.8 – 23.5) | |
| $p$ | 0.41 (0.18 – 0.68) | |
| $\delta$ | 52 | |
| $p_{Dx}^{\left( pu \right)}$ | 0.84 (0.75 – 0.89) | |
| $p_{Dx}^{\left( pr \right)}$ | 0.19 (0.10 – 0.29) | |
| $\tau$ | 2 | |
| $\epsilon^{\left( pu \right)}$ | 0.38 (0.12 – 0.65) | |
| $\epsilon^{\left( pr \right)}$ | 1.5 (0.5 – 2.9) | |
| $\mu$ | 1/69 | |

| Country: Sri Lanka | | |
| --- | --- | --- |
| Parameter Symbol | Value | |
| $\beta$ | 12 (6 – 18) | |
| $u$ | 0.19 (0.05 – 0.32) | |
| $v$ | 0.94 (0.68 – 1.07) | |
| $w$ | 0.001 (0.0001 – 0.0038) | |
| $k_{1}$ | 1.42 (1.26 – 1.57) | |
| $y$ | 0.27 | |
| $c$ | 0.027 (0.021 – 0.034) | |
| $\mu_{TB}$ | 0.09 (0.02 – 0.16) (for normal-BMI) | ${2*\mu}_{TB}$(for low-BMI)^2^ |
| $\mu_{TB}^{Tx}$ | 0.098 (for normal-BMI) | 0.15 (for low-BMI) |
| $\sigma$ | 0.99 (0.09 – 1.6) | |
| $h$ | 0.34 (0.11 – 0.72) | |
| $\rho^{\left( lo \right)}$ | 0.030 (0.024 – 0.04) (for normal-BMI) | |
| $\rho^{\left( hi \right)}$ | 0.13 (0.11 – 0.17) (for normal-BMI) | |
| $\rho$ | 0.0014 (0.0011 – 0.0018) (for normal-BMI) | |
| $z$ | 0.75 | |
| $s$ | 0.5 | |
| $\gamma$ | 7.7 (2.8 – 9.9) | |
| $\tilde{\gamma}$ | 14.5 (4.2 – 23.4) | |
| $p$ | 0.46 (0.19 – 0.68) | |
| $\delta$ | 52 | |
| $p_{Dx}^{\left( pu \right)}$ | 0.83 (0.75 – 0.89) | |
| $p_{Dx}^{\left( pr \right)}$ | 0.20 (0.11 – 0.29) | |
| $\tau$ | 2 | |
| $\epsilon^{\left( pu \right)}$ | 0.35 (0.12 – 0.66) | |
| $\epsilon^{\left( pr \right)}$ | 1.4 (0.5 – 2.8) | |
| $\mu$ | 1/76 | |

| Country: Thailand | | |
| --- | --- | --- |
| Parameter Symbol | Value | |
| $\beta$ | 17 (7 – 27) | |
| $u$ | 0.15 (0.02 – 0.36) | |
| $v$ | 0.91 (0.67 – 1.08) | |
| $w$ | 0.0024 (0.0003 – 0.0055) | |
| $k_{1}$ | 1.75 (1.56 – 1.95) | |
| $y$ | 0.30 | |
| $c$ | 0.028 (0.021 – 0.035) | |
| $\mu_{TB}$ | 0.23 (0.08 – 0.45) (for normal-BMI) | ${2*\mu}_{TB}$(for low-BMI)^2^ |
| $\mu_{TB}^{Tx}$ | 0.098 (for normal-BMI) | 0.15 (for low-BMI) |
| $\sigma$ | 0.57 (0.04 – 1.5) | |
| $h$ | 0.36 (0.11 – 0.72) | |
| $\rho^{\left( lo \right)}$ | 0.032 (0.024 – 0.04) (for normal-BMI) | |
| $\rho^{\left( hi \right)}$ | 0.14 (0.11 – 0.17) (for normal-BMI) | |
| $\rho$ | 0.0014 (0.0011 – 0.0018) (for normal-BMI) | |
| $z$ | 0.75 | |
| $s$ | 0.5 | |
| $\gamma$ | 7.6 (2.8 – 9.9) | |
| $\tilde{\gamma}$ | 15.4 (4.3 – 23.5) | |
| $p$ | 0.57 (0.34 – 0.73) | |
| $\delta$ | 52 | |
| $p_{Dx}^{\left( pu \right)}$ | 0.82 (0.75 – 0.89) | |
| $p_{Dx}^{\left( pr \right)}$ | 0.20 (0.10 – 0.30) | |
| $\tau$ | 2 | |
| $\epsilon^{\left( pu \right)}$ | 0.36 (0.12 – 0.64) | |
| $\epsilon^{\left( pr \right)}$ | 1.3 (0.5 – 2.9) | |
| $\mu$ | 1/79 | |

| Country: Timor-Leste | | |
| --- | --- | --- |
| Parameter Symbol | Value | |
| $\beta$ | 26 (16 – 29) | |
| $u$ | 0.04 (0.01 – 0.11) | |
| $v$ | 0.92 (0.67 – 1.1) | |
| $w$ | 0.0032 (0.0003 – 0.0069) | |
| $k_{1}$ | 1.81 (1.69 – 1.95) | |
| $y$ | 0.30 | |
| $c$ | 0.025 (0.021 – 0.033) | |
| $\mu_{TB}$ | 0.20 (0.07 – 0.38) (for normal-BMI) | ${2*\mu}_{TB}$(for low-BMI)^2^ |
| $\mu_{TB}^{Tx}$ | 0.098 (for normal-BMI) | 0.15 (for low-BMI) |
| $\sigma$ | 0.37 (0.03 – 1.2) | |
| $h$ | 0.52 (0.14 – 0.74) | |
| $\rho^{\left( lo \right)}$ | 0.031 (0.024 – 0.04) (for normal-BMI) | |
| $\rho^{\left( hi \right)}$ | 0.14 (0.11 – 0.17) (for normal-BMI) | |
| $\rho$ | 0.0015 (0.0011 – 0.0019) (for normal-BMI) | |
| $z$ | 0.75 | |
| $s$ | 0.5 | |
| $\gamma$ | 3.6 (1.1 – 9.5) | |
| $\tilde{\gamma}$ | 5.5 (1.2 – 20.2) | |
| $p$ | 0.39 (0.16 – 0.68) | |
| $\delta$ | 52 | |
| $p_{Dx}^{\left( pu \right)}$ | 0.82 (0.75 – 0.89) | |
| $p_{Dx}^{\left( pr \right)}$ | 0.17 (0.10 – 0.30) | |
| $\tau$ | 2 | |
| $\epsilon^{\left( pu \right)}$ | 0.34 (0.12 – 0.65) | |
| $\epsilon^{\left( pr \right)}$ | 1.3 (0.5 – 2.8) | |
| $\mu$ | 1/68 | |

# Model calibration

The model was first calibrated to pre-COVID conditions using incidence (2019), notification (2019), mortality (2019), relative risk of TB among low-BMI population, prevalence of under nutrition, and LTBI prevalence of the respective countries. In particular, for each of the data elements shown in Table 1, we constructed a likelihood function using log-normal distributions to model all population rates (such as annual incidence) and beta distributions to model all parameters (such as the proportion of the population have undernutrition). For each data element, we chose the corresponding distributional parameters to give the same 2.5^th^, 50^th^ and 97.5^th^ percentiles as in the data. For example, to construct the likelihood function $L_{incd}\left( . \right)$ corresponding to incidence, we defined the objective function:

$$ssq\left( \mu,\sigma\right)={[iCDF\left( 0.025 \right|\mu,\sigma)-d_{1}]}^{2}+{[iCDF\left( 0.50 \right|\mu,\sigma)-d_{2}]}^{2}+{[iCDF\left( 0.975 \right|\mu,\sigma)-d_{3}]}^{2},$$

Where iCDF is the inverse cumulative density function for given log-normal parameters $\mu,\sigma$; and $d_{1}, d_{2},d_{3}$ denote respectively the lower, central and upper estimates in the data. We then determined values of $\mu,\sigma$ in order to minimise $ssq\left( \mu, \sigma\right).$

Repeating this process for other data elements, we then evaluated the overall likelihood as a product of these individual likelihoods. Finally, we assumed uniform distributions on all model parameters, within plausible ranges shown in Table S1 and S2. We defined the posterior density as a product of all such prior distributions, and the likelihood term. In practice we worked with the log-posterior density, thus taking a sum of all individual log-likelihood terms.

To sample from the posterior density, we used adaptive Markov Chain Monte Carlo (MCMC)^19^. In brief, during an MCMC chain, this approach uses the covariance matrix from already-sampled parameter values to inform the proposal distribution at any given step. More advanced methods have since been developed^20^: nonetheless, this approach has the great benefit of simplicity, and – as illustrated by figures in section 4,’ *Posterior distributions of calibrated parameters*’ – performs adequately in creating well-mixed posterior samples that show good agreement with the data. We performed each MCMC for 200,000 iterations. To remove the ‘burn-in’, we discarded the first 50% of these samples. We then ‘thinned’ the sample by selecting every 400^th^ sample, ultimately to yield 250 samples from the posterior density.

Finally, to capture the effects of COVID-related disruptions, the model was matched to quarterly notification data from Q1 2020 onwards, using the 250 samples produced by calibration to pre-pandemic data, and using the same methodology as used by WHO to generate TB burden estimates^21^. Figures S2 and S3 show the calibration results fitted with the pre-COVID data and Figure S4 shows the post-COVID notification. Figure S11 shows modelled projections in the example of India for different sizes (i.e. number of samples) of the posterior sample, illustrating that our choice of 250 samples yields projections that are stable with respect to the sample size.

**
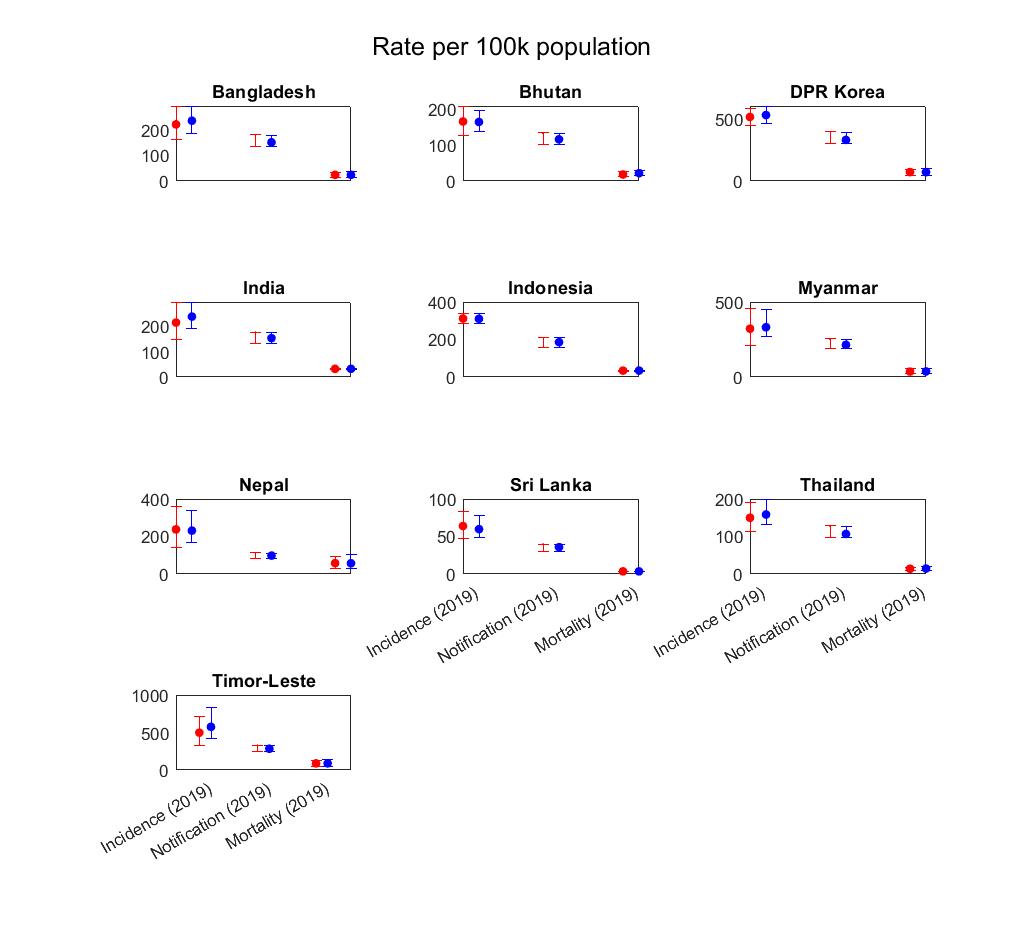
**

**Figure S2. Model agreement with calibration targets** for incidence, mortality and notifications, for each country in the SEA Region. Points in red show data, points in blue show model simulations, and vertical bars show 95% uncertainty intervals.

**
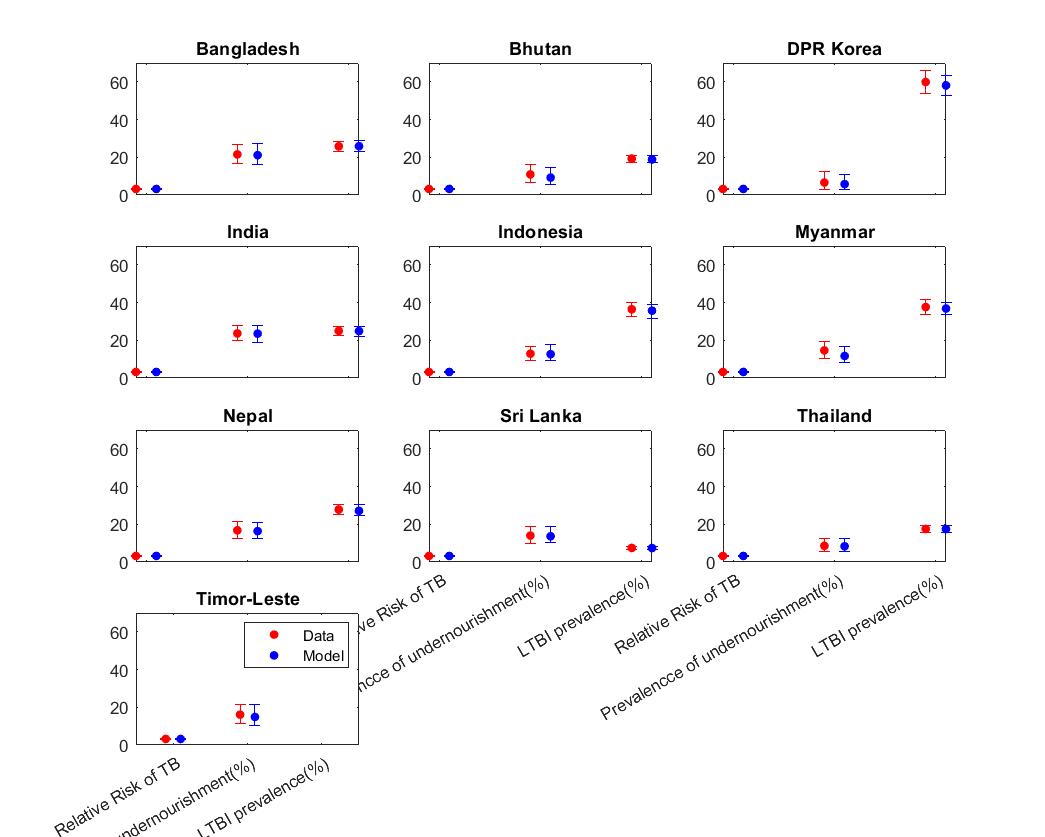
**

**Figure S3. Model agreement with calibration targets** for the relative risk of TB amongst those with undernutrition compared to those with normal BMI; prevalence of undernutrition; and prevalence of TB infection (LTBI). Points in red show data, points in blue show model simulations, and vertical bars show 95% uncertainty intervals.

**
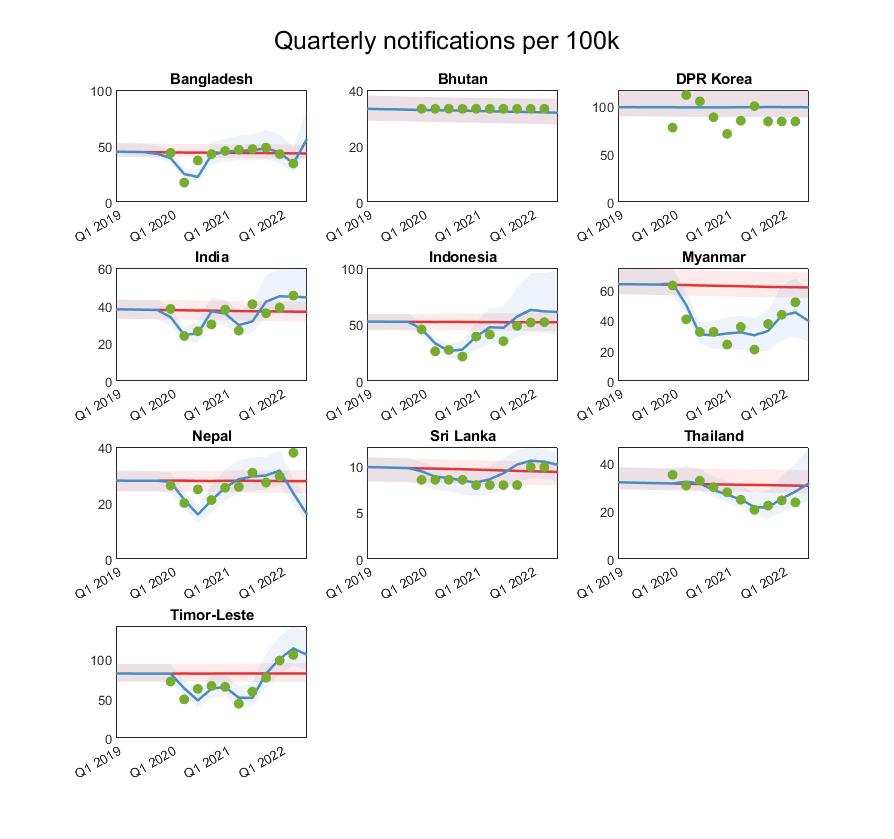
**

**Figure S4.** From Q1 2020 onwards, the model is fitted to quarterly notification data. Green points show reported data while the blue-line shows the model-fitted notification.

**
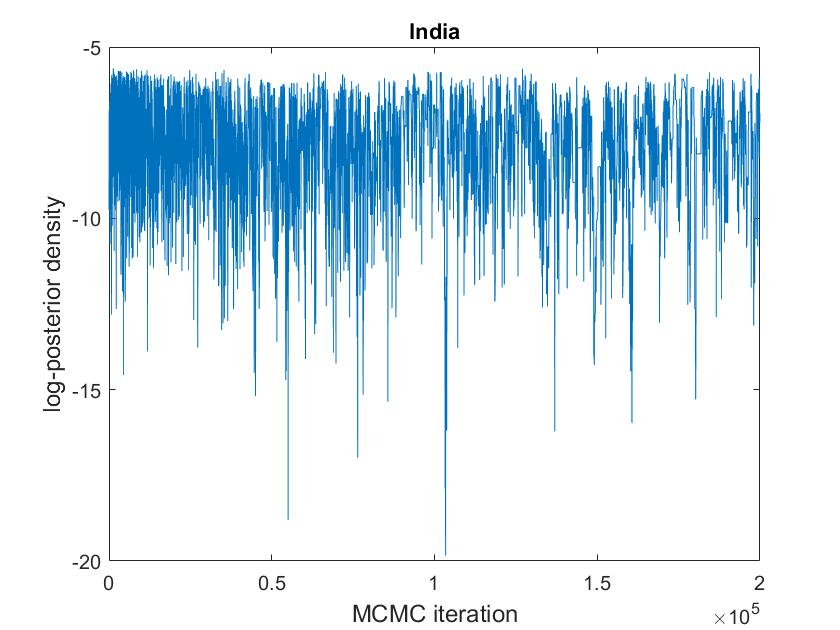
**

**Figure S5**. Trace of samples from the posterior density for India (as an example)

1. Posterior distributions of calibrated parameters


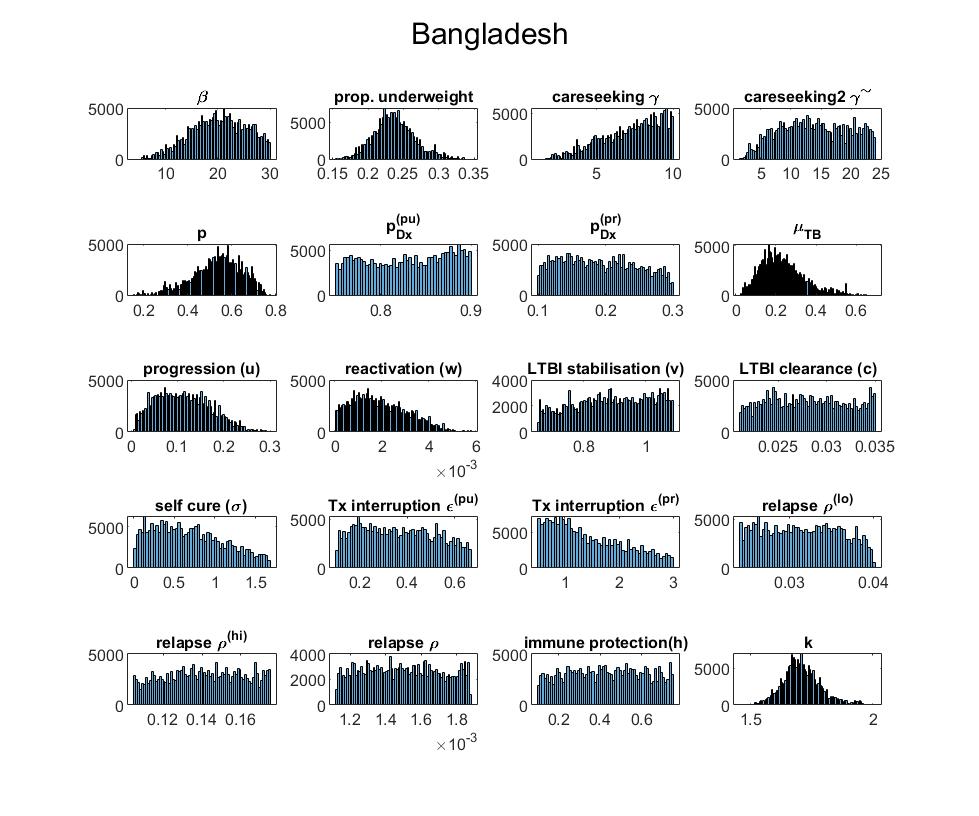


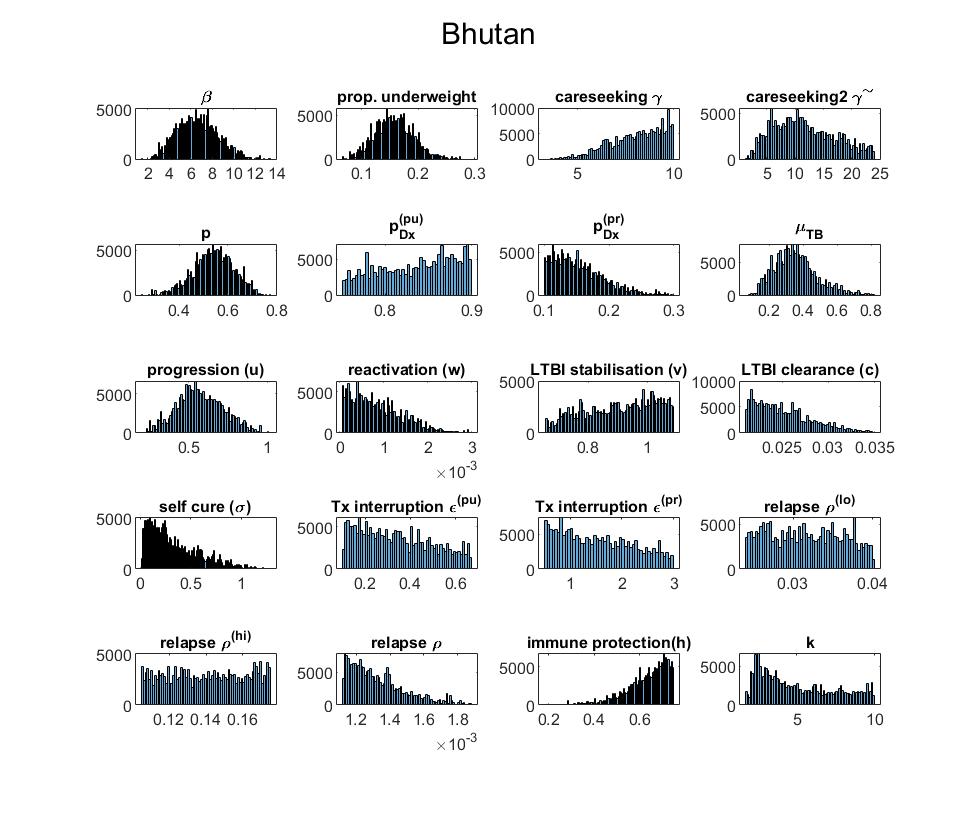


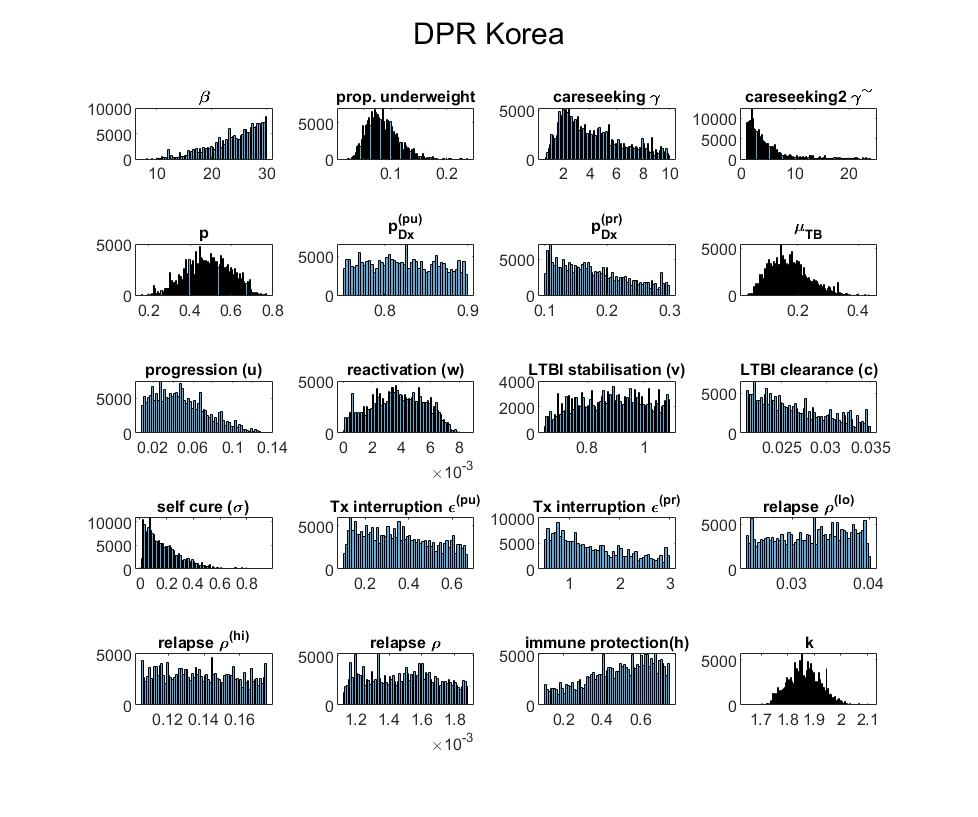


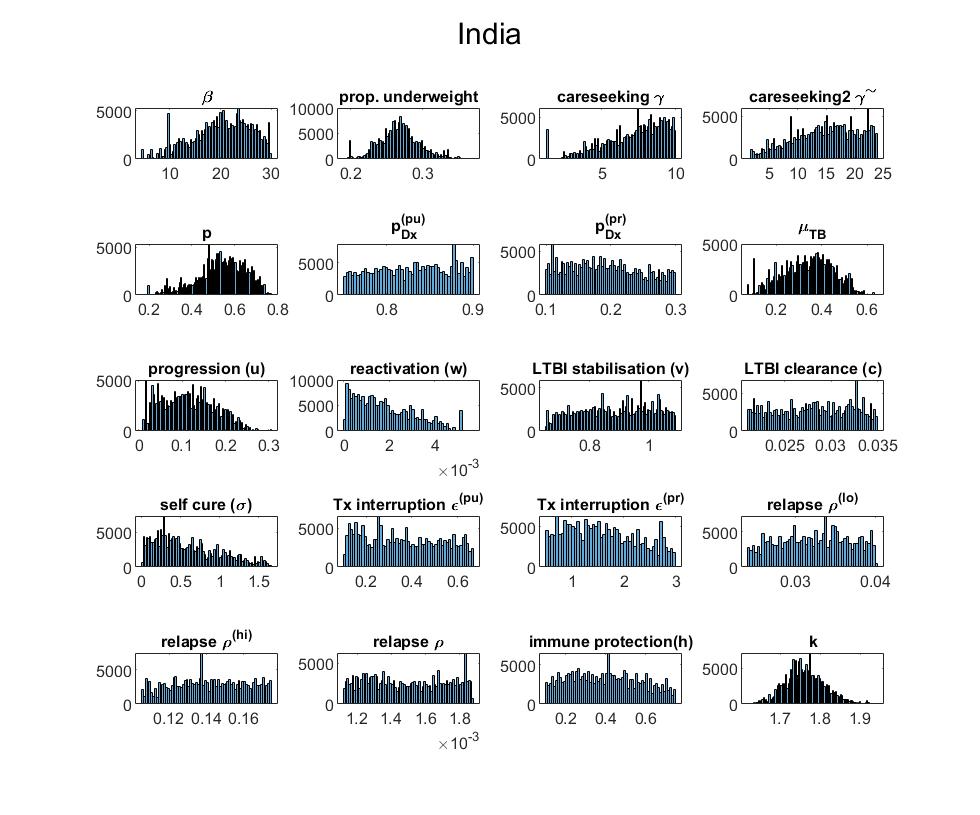


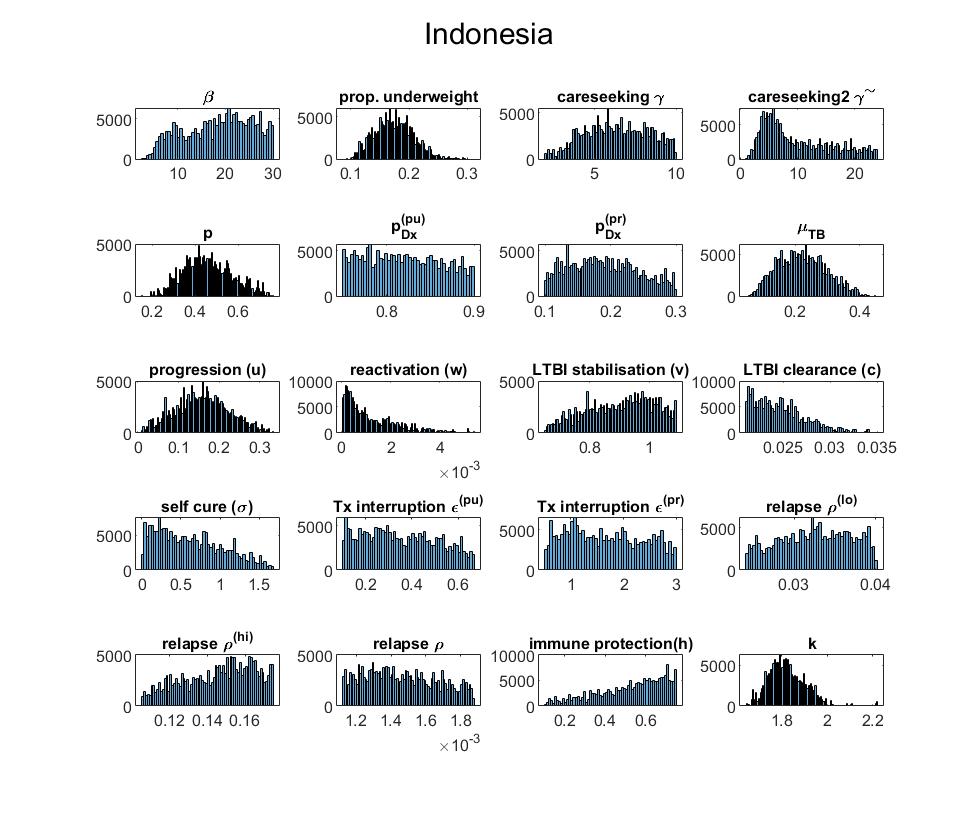


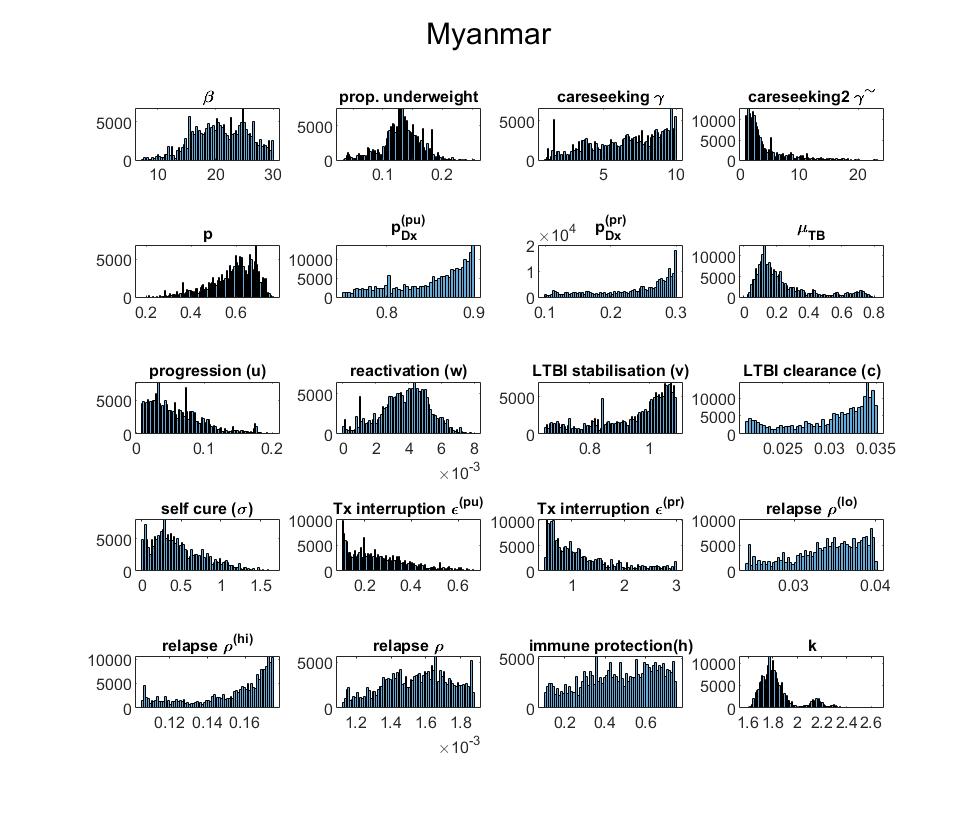


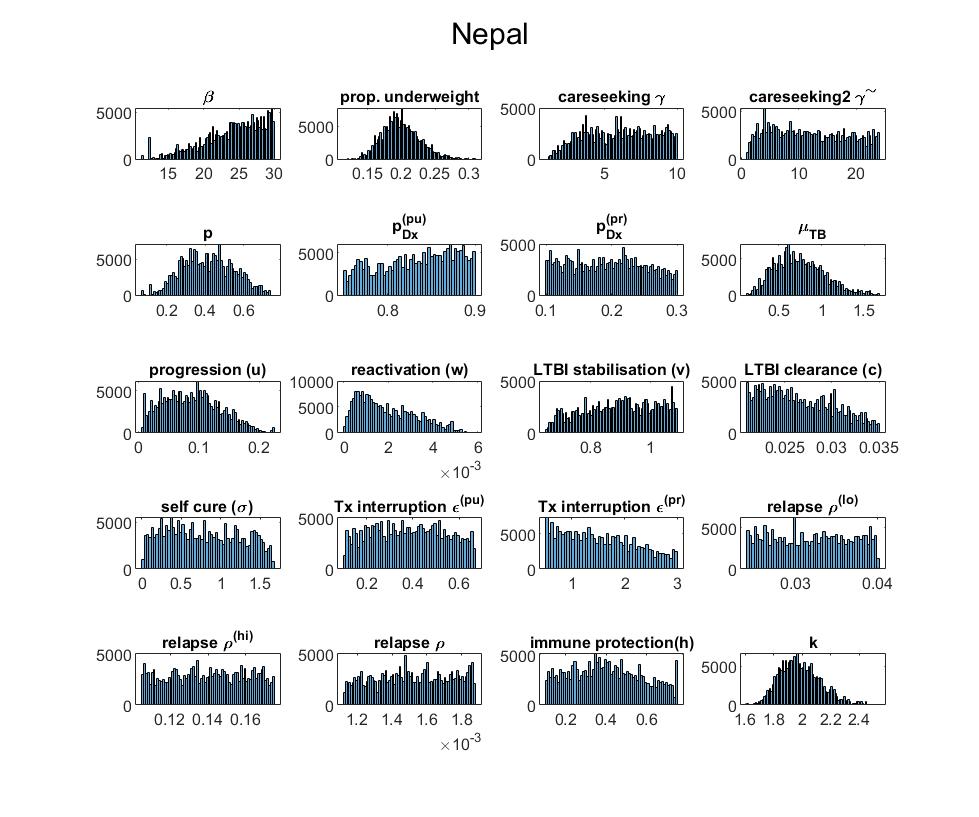


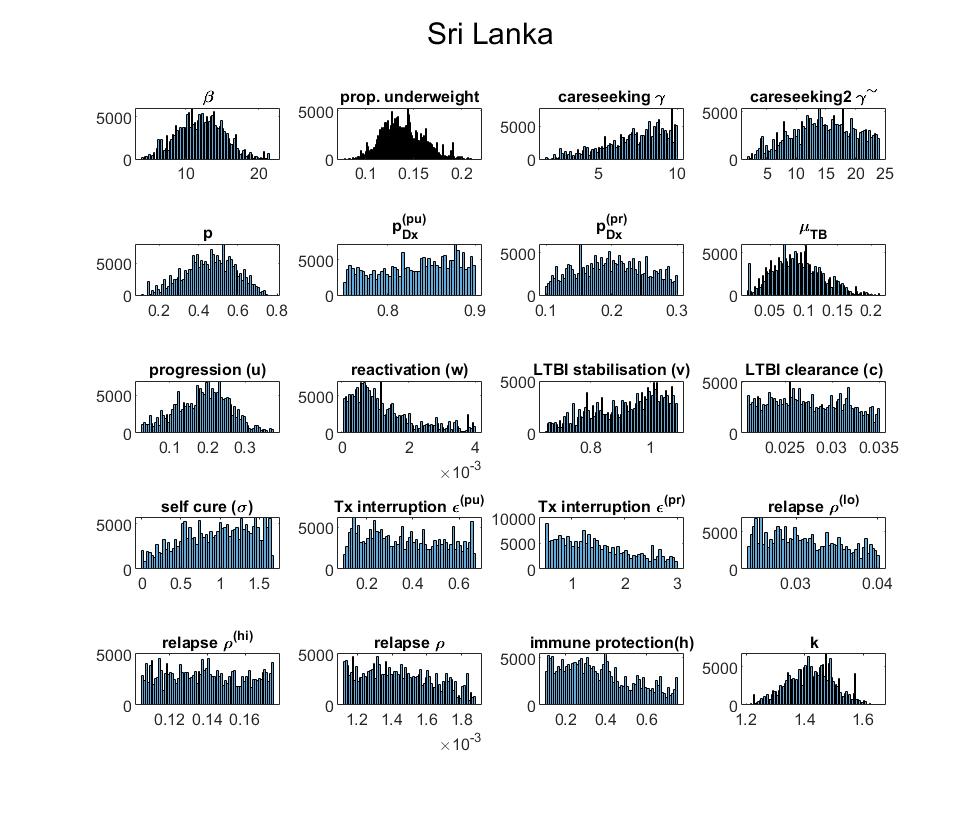


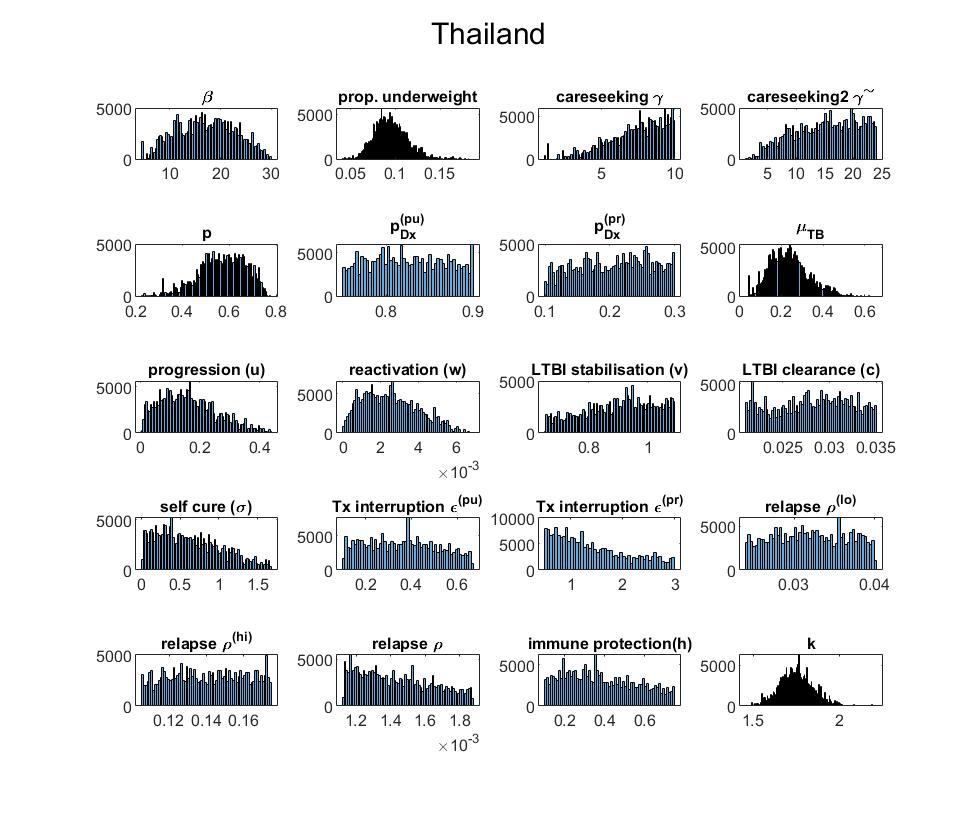


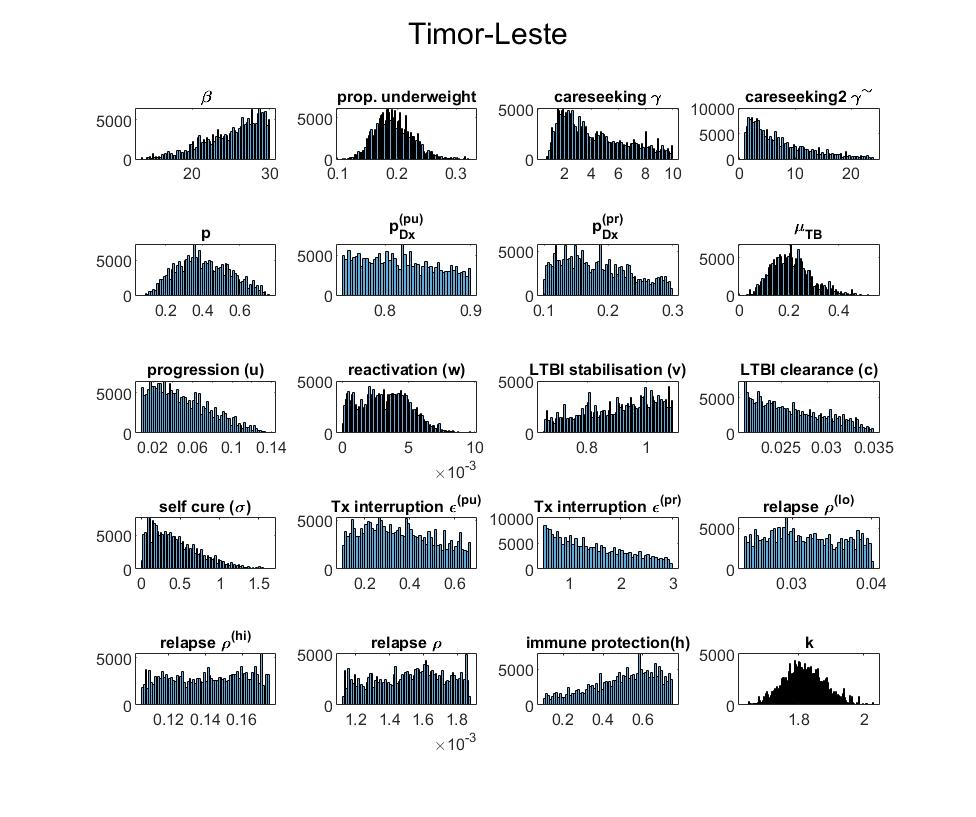


**Figure S6.** Posterior distribution of model parameters

**
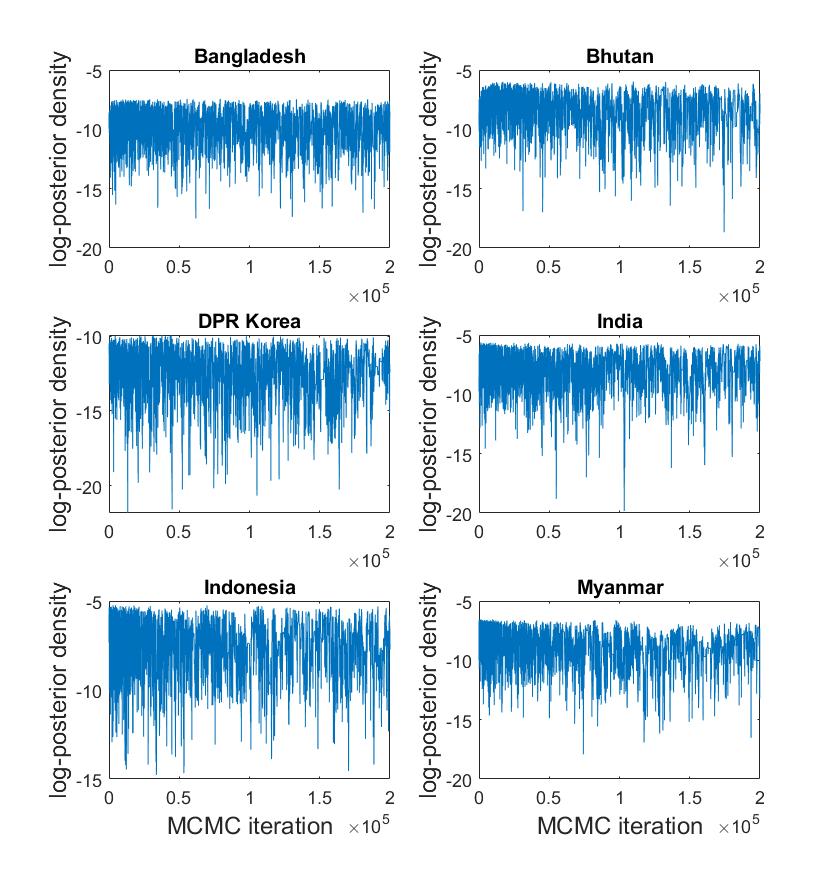
**

**
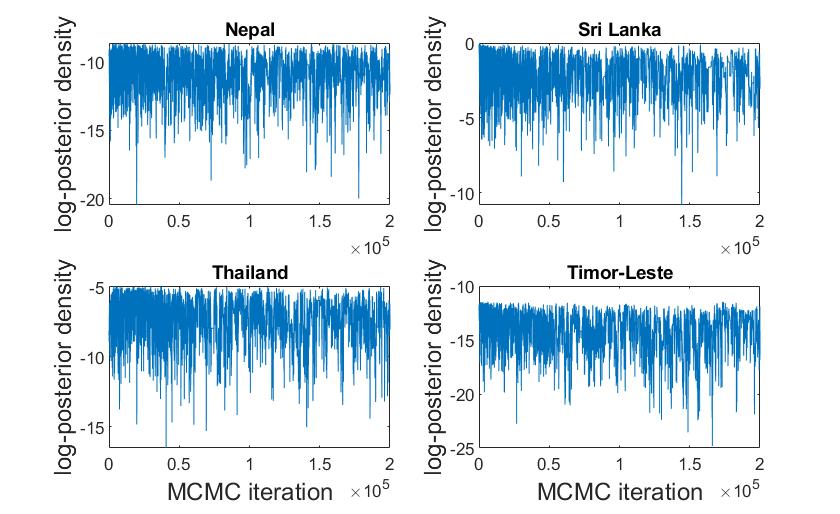
**

**Figure S7.** Trace of samples from the posterior density for all studied countries (For India see figure S5)

# Modelling interventions

As described in the main text, we simulated two interventions: (i) nutritional rehabilitation amongst all household contacts of index TB cases, regardless of pre-existing nutritional status, and (ii) amongst all those in the population with undernutrition, providing nutritional rehabilitation to 30% per year.

Modelling household contacts is challenging in compartmental models, which do not lend themselves to capturing such detailed levels of contact structure. To address this challenge, we drew from a previously developed methodology, applied in the context of TB preventive therapy in the Region^22^. This methodology is described in detail elsewhere (Supplementary information^22^) but, in brief, involved three steps in the current study: first, estimating the proportion of incident TB in a given year that would be averted through the *direct* effects of nutritional rehabilitation (i.e. the 40% reduction in incidence amongst receiving this intervention that was observed in the RATIONS trial^6^); second, adjusting the population-level rates of reactivation and progression in order to capture this same direct effect; and third, simulating the full transmission model with these adjusted rates in effect. ^1^We assumed this intervention to be rolled out to the whole country’s population between 2023 and 2025. In order to perform the second step, we adjusted only the reactivation and progression parameters of those with undernutrition (i.e. the right-hand column of Figure S1A).

To model nutritional rehabilitation in the general population, we simply modelled a transition from the right-hand column of Figure S1A to the middle column at a per-capita rate of $0.15 yr^{-1}$, chosen to correspond to 30% of people with low-BMI being rehabilitated each year. We assumed further that in the general population, rehabilitation amongst those with low BMI would lead to the same incidence reductions as amongst undernourished contacts in the RATIONS trial, i.e. 40%^6^ .

Finally, as described in the main text, we also modelled programmatic improvements alongside nutritional interventions: in particular, to model a 30% reduction in the average delay to careseeking, we modelled a 43% increase in the parameters $\gamma$ and $\tilde{\gamma}$ (see Table S1 and Figure S1B), again assumed to occur in a linear way between 2023 and 2025. To model an increase to 90% in the proportion of private providers notifying TB, we assumed that such providers can be counted together with the public sector, and thus modelled an increase in the parameter $p$ (see Table S1 and Figure S1B) from its calibrated value to a value of 0.9, again assuming this increase to occur in a linear way between 2023 and 2025.


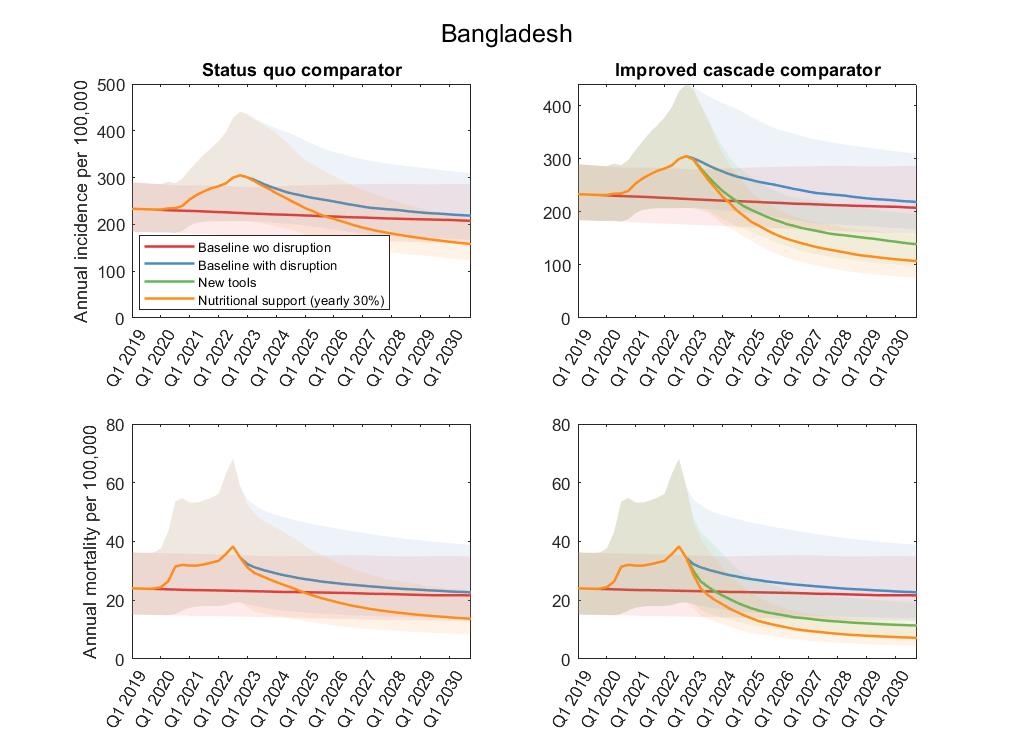


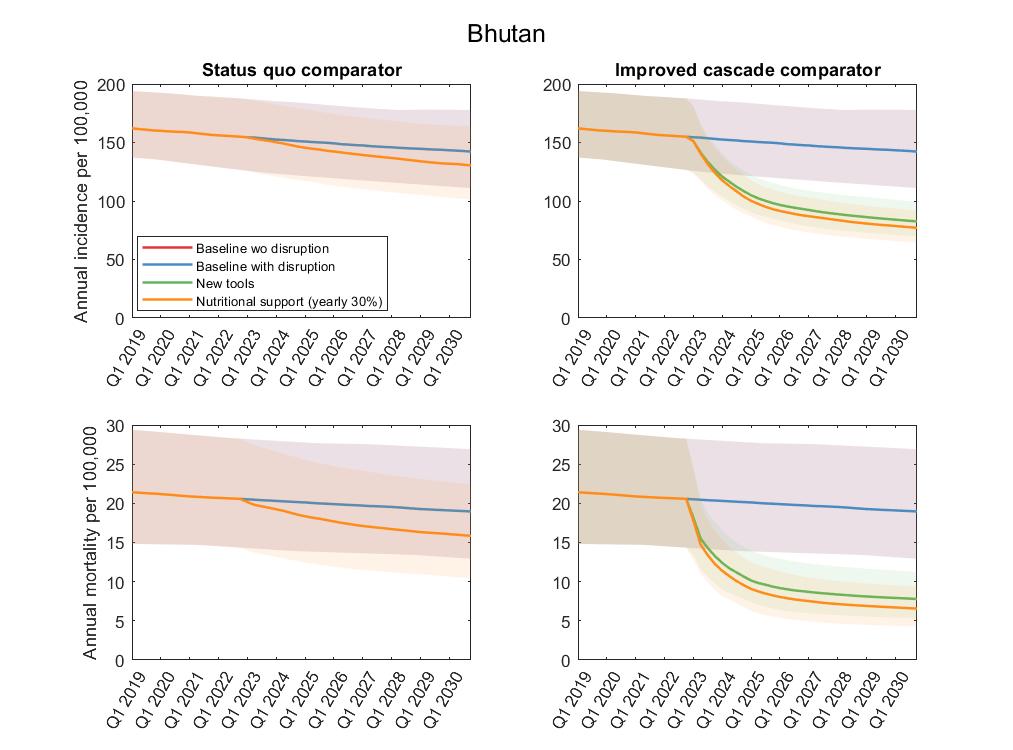


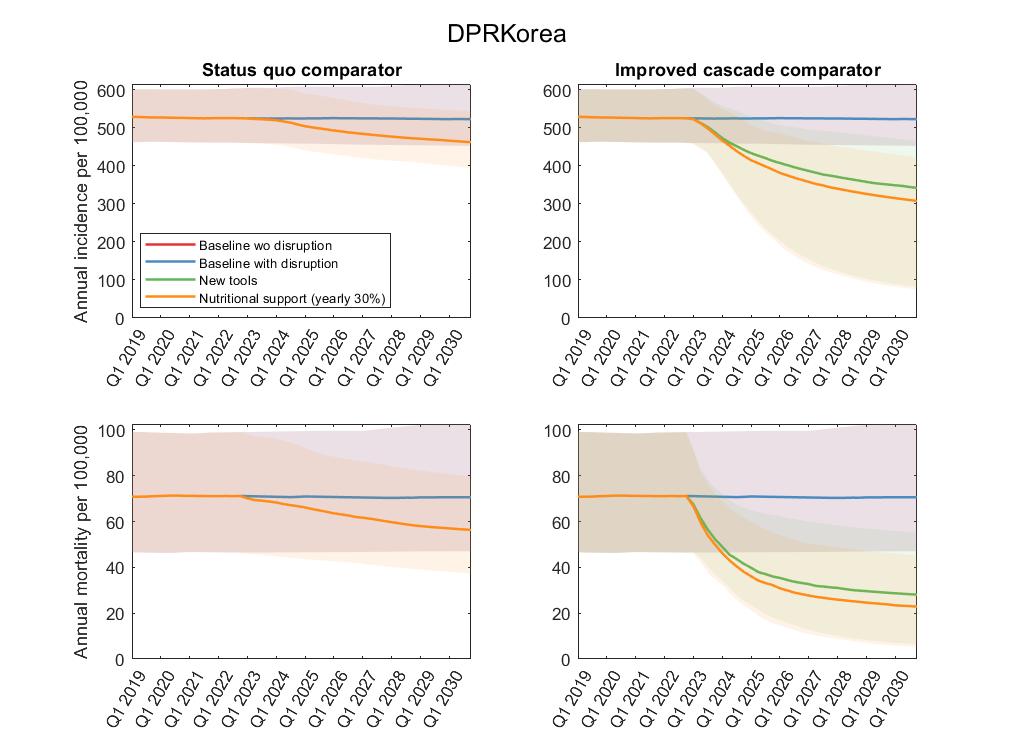


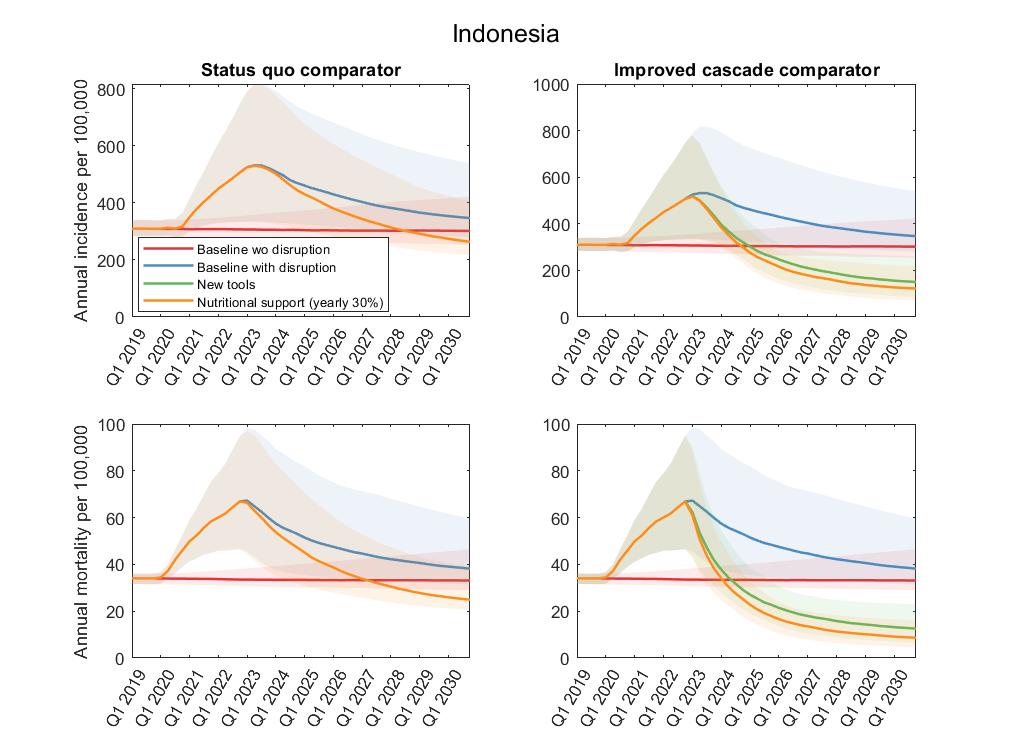


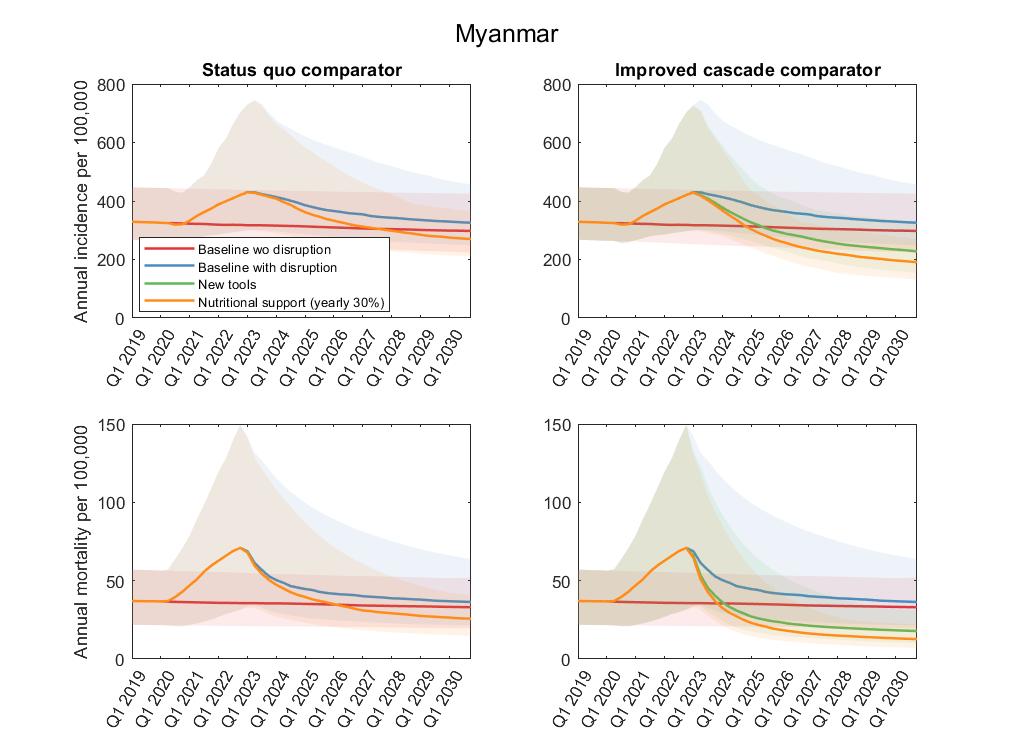


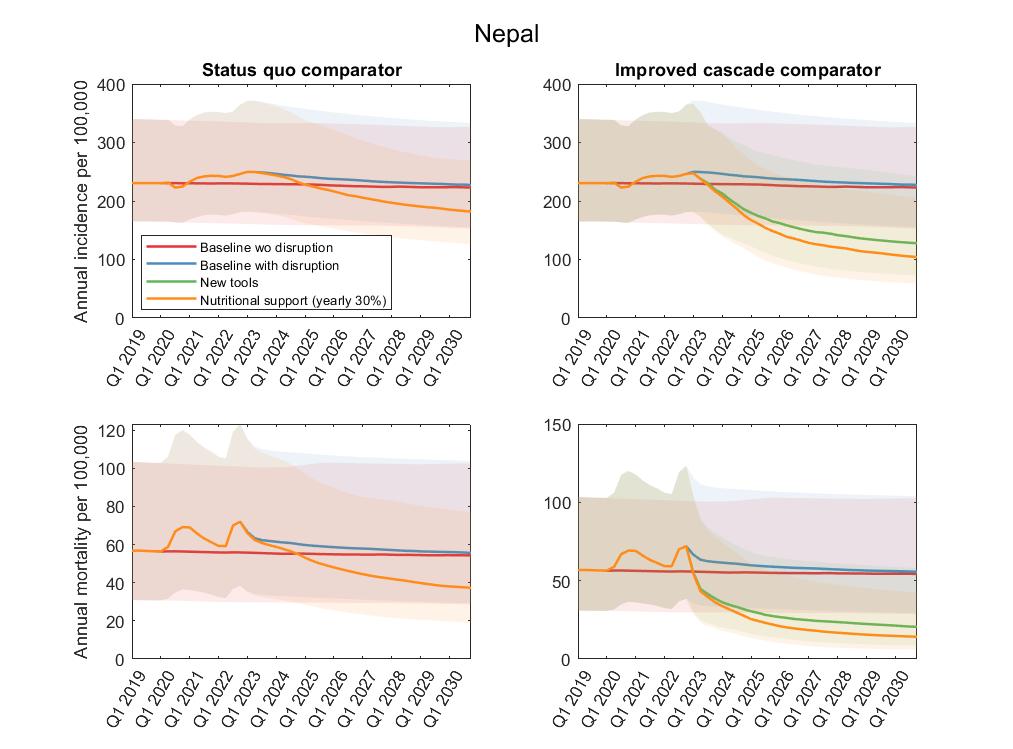


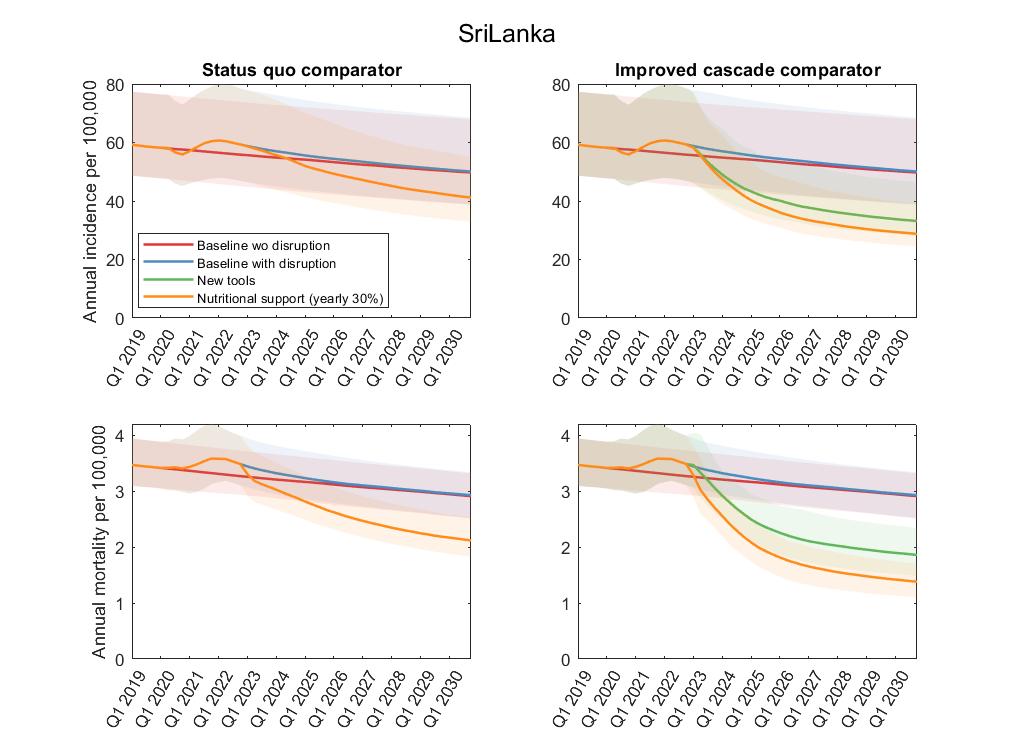


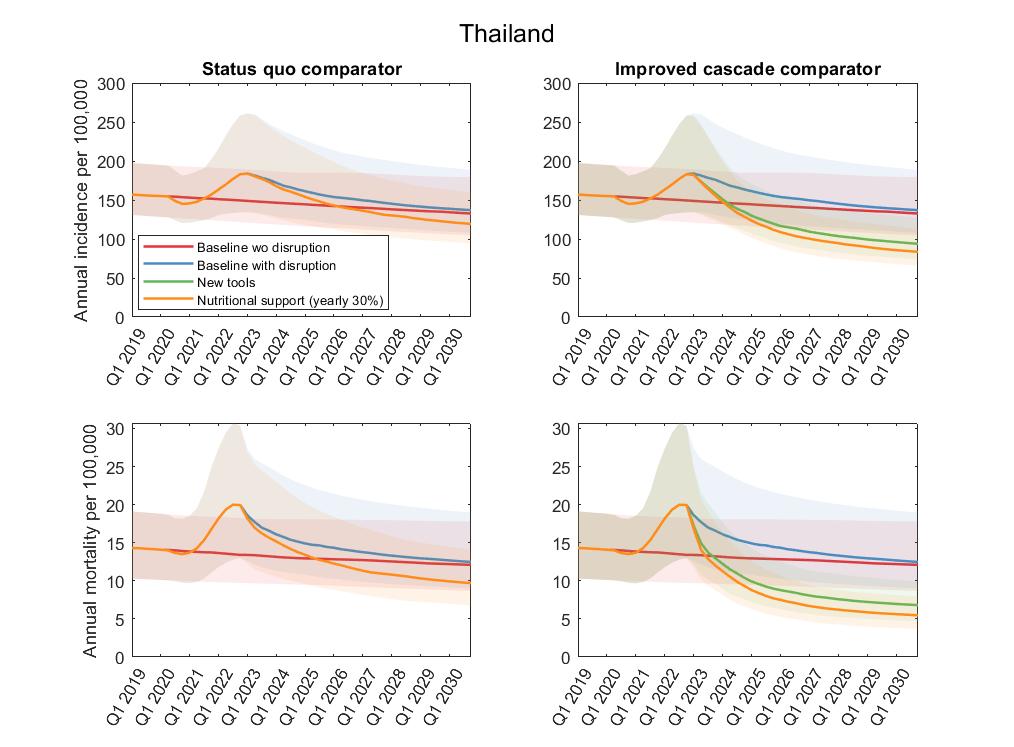


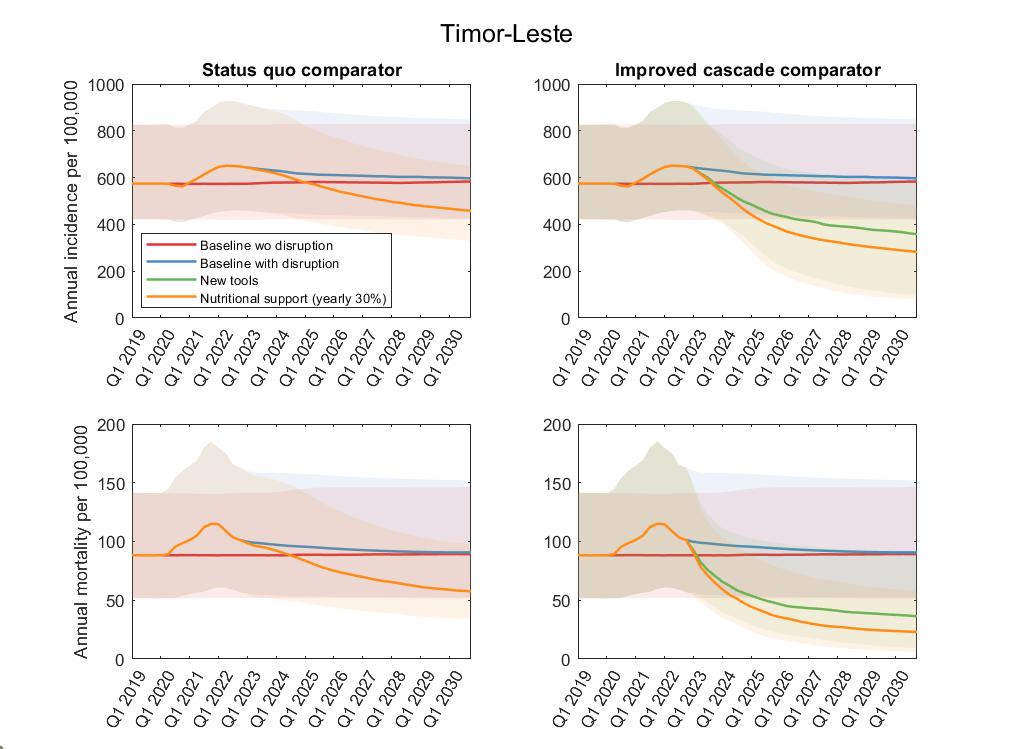


**Figure S8. Impact of nutritional interventions for different countries (except India, which is shown in Figure 2 in the main text).** Here, figures in the upper row show projections for incidence, taking account of COVID-related disruptions in that country, while figures in the lower row show projections for mortality. The left-hand column shows the effect of nutritional interventions acting alone, while the right-hand column shows the effect of these interventions when acting in combination with measures to improve the TB care cascade.

# Sensitivity analyses

In the main text, we modelled the potential impact of interventions aimed at nutritionally rehabilitating 30% of people with undernutrition, in the general community (Figure 2). In the example of India, Figure S9 shows sensitivity analysis with additional scenarios of 5%, 10%, 20% and 40% coverage.


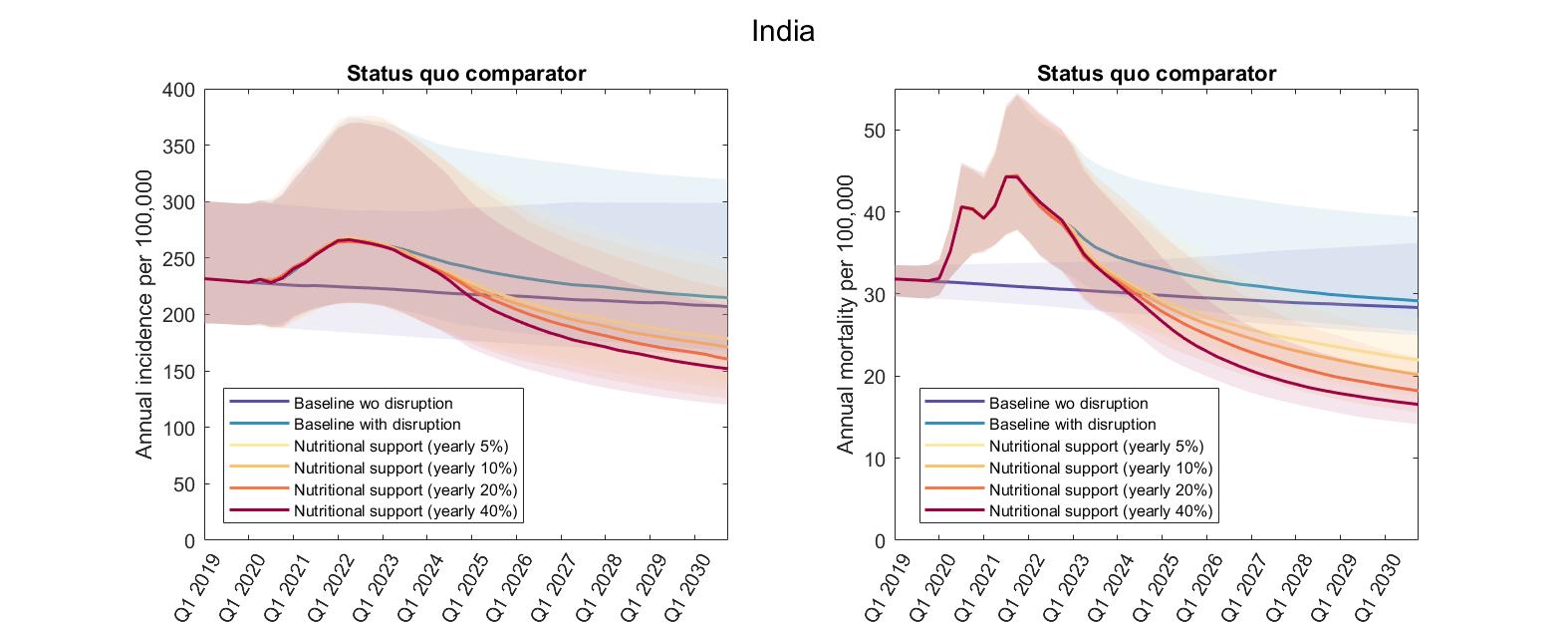


**Figure S9**. Impact of nutritional interventions in the general community, under three different coverage scenarios for annual coverage of nutritional rehabilitation amongst those with BMI < 18.5 kg/m2.

The prevalence of underweight among adults is around 23.6% [19.9 – 27.6] in India (See Table 1). Figure S10 shows alternative scenarios of impact of nutritionally rehabilitating 30% of those with undernutrition in the general population, when undernutrition accounts for 10% [8.5 – 11.5] and 50% [25.5 – 34.5] of the country population.


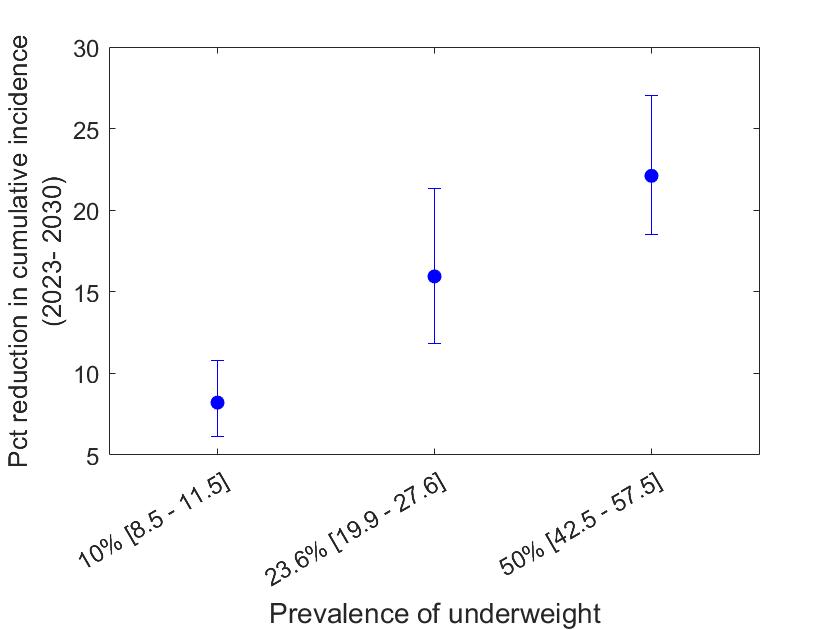


**Figure S10.** Percentage reduction of cumulative incidence in India (2003 - 2030) by intervening 30% of underweight population under three scenarios for the baseline prevalence of underweight.


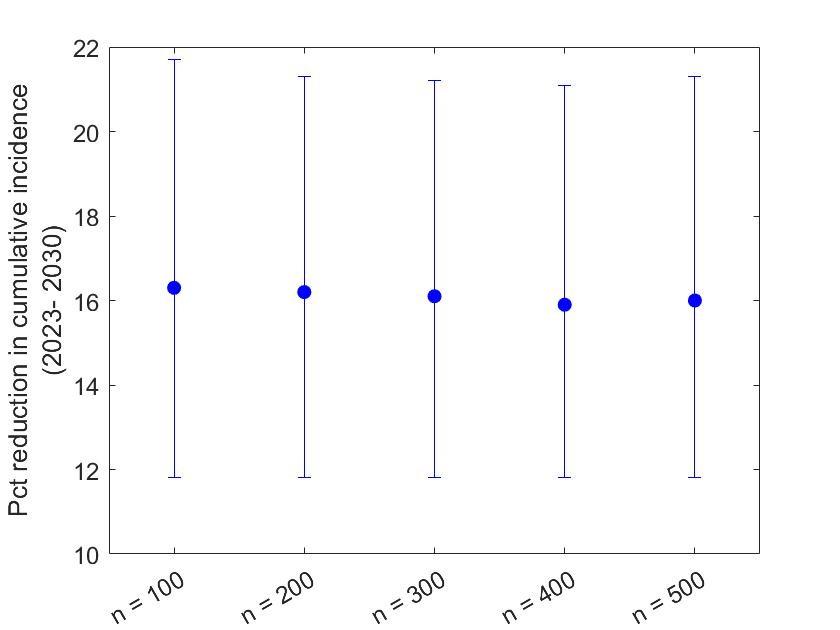


**Figure S11**. Sensitivity analysis to the size of the posterior sample. Shown are estimates for the impact in India (percent cases averted by the community-based intervention shown in Fig.2) when using different numbers of samples ($n$) from the posterior density.

# References

1 Bhargava A, Bhargava M, Meher A, *et al.* Nutritional support for adult patients with microbiologically confirmed pulmonary tuberculosis: outcomes in a programmatic cohort nested within the RATIONS trial in Jharkhand, India. *Lancet Glob Health* 2023; **11**: e1402–11.

2 Bhargava A, Bhargava M. Tuberculosis deaths are predictable and preventable: Comprehensive assessment and clinical care is the key. *J Clin Tuberc Other Mycobact Dis* 2020; **19**. DOI:10.1016/j.jctube.2020.100155.

3 World Health Organization. Global Tuberculosis Report 2023. 2023.

4 Menzies NA, Wolf E, Connors D, *et al.* Progression from latent infection to active disease in dynamic tuberculosis transmission models: a systematic review of the validity of modelling assumptions. Lancet Infect Dis. 2018; **18**: e228–38.

5 World Health Organization. Global Tuberculosis Report 2021. 2021.

6 Bhargava A, Bhargava M, Meher A, *et al.* Nutritional supplementation to prevent tuberculosis incidence in household contacts of patients with pulmonary tuberculosis in India (RATIONS): a field-based, open-label, cluster-randomised, controlled trial. *The Lancet* 2023; **402**: 627–40.

7 Emery JC, Richards AS, Dale KD, *et al.* Self-clearance of Mycobacterium tuberculosis infection: Implications for lifetime risk and population at-risk of tuberculosis disease. *Proceedings of the Royal Society B: Biological Sciences* 2021; **288**. DOI:10.1098/rspb.2020.1635.

8 India TB Report 2023. 2023 https://tbcindia.gov.in/showfile.php?lid=3680 (accessed Jan 25, 2024).

9 Tiemersma EW, van der Werf MJ, Borgdorff MW, Williams BG, Nagelkerke NJD. Natural history of tuberculosis: Duration and fatality of untreated pulmonary tuberculosis in HIV negative patients: A systematic review. PLoS One. 2011; **6**. DOI:10.1371/journal.pone.0017601.

10 Andrews JR, Noubary F, Walensky RP, Cerda R, Losina E, Horsburgh CR. Risk of progression to active tuberculosis following reinfection with Mycobacterium tuberculosis. *Clinical Infectious Diseases* 2012; **54**: 784–91.

11 Thomas A, Gopi P, Santha T, *et al.* Predictors of relapse among pulmonary tuberculosis patients treated in a DOTS programme in South India. *Int J Tuberc Lung Dis* 2005; **9**: 556–61.

12 Romanowski K, Balshaw RF, Benedetti A, *et al.* Predicting tuberculosis relapse in patients treated with the standard 6-month regimen: An individual patient data meta-analysis. *Thorax* 2019; **74**: 291–7.

13 Menzies D, Benedetti A, Paydar A, *et al.* Effect of duration and intermittency of rifampin on tuberculosis treatment outcomes: A systematic review and meta-analysis. PLoS Med. 2009; **6**. DOI:10.1371/journal.pmed.1000146.

14 Weis S, Slocum P, Blais F, *et al.* The effect of directly observed therapy on the rates of drug resistance and relapse in tuberculosis. *N Engl J Med* 1994; **330**: 1179–84.

15 Guerra-Assuncąõ JA, Houben RMGJ, Crampin AC, *et al.* Recurrence due to relapse or reinfection with mycobacterium tuberculosis: A whole-genome sequencing approach in a large, population-based cohort with a high HIV infection prevalence and active follow-up. *Journal of Infectious Diseases* 2015; **211**: 1154–63.

16 Khan A, Sterling TR, Reves R, Vernon A, Horsburgh CR. Lack of weight gain and relapse risk in a large tuberculosis treatment trial. *Am J Respir Crit Care Med* 2006; **174**: 344–8.

17 Subbaraman R, Nathavitharana RR, Satyanarayana S, *et al.* The Tuberculosis Cascade of Care in India’s Public Sector: A Systematic Review and Meta-analysis. *PLoS Med* 2016; **13**. DOI:10.1371/journal.pmed.1002149.

18 The World Bank. India demographic data. . 2021 https://data.worldbank.org/country/india (accessed Oct 26, 2023).

19 Haario H, Saksman E, Tamminen J. An adaptive Metropolis algorithm. *Bernoulli* 2001; **7**: 223–42.

20 Brooks S, Gelman A, Jones GL, Meng X-L, editors. Handbook of Markov Chain Monte Carlo. Chapman & Hall/CRC, 2011.

21 Bastard M, Arinaminpathy N, Dodd P J, Timimi H, Dean A, Floyd K. Methods used by WHO to estimate the global burden of TB disease. 2023.

22 Mandal S, Bhatia V, Sharma M, Mandal PP, Arinaminpathy N. The potential impact of preventive therapy against tuberculosis in the WHO South-East Asian Region: A modelling approach. *BMC Med* 2020; **18**. DOI:10.1186/s12916-020-01651-5.
